# Real-Time In Situ Monitoring of CO_2_ Electroreduction in the Liquid and Gas Phases by Coupled Mass Spectrometry and Localized Electrochemistry

**DOI:** 10.1021/acscatal.2c00609

**Published:** 2022-05-10

**Authors:** Guohui Zhang, Youxin Cui, Anthony Kucernak

**Affiliations:** Department of Chemistry, Imperial College London, London SW7 2AZ, United Kingdom

**Keywords:** CO_2_ reduction reaction, correlative electrochemical measurements, gas diffusion electrode, mass spectrometry, ultramicroelectrode

## Abstract

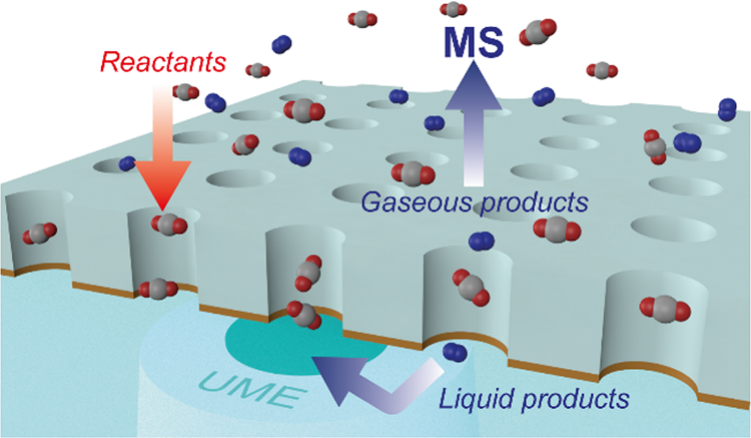

The mechanism and dynamics of the CO_2_ reduction reaction (CO_2_RR) remain poorly understood, which is largely caused by mass transport limitations and lack of time-correlated product analysis tools. In this work, a custom-built gas accessible membrane electrode (GAME) system is used to comparatively assess the CO_2_RR behavior of Au and Au−Cu catalysts. The platform achieves high reduction currents (∼ – 50 mA cm^–2^ at 1.1 V vs RHE) by creating a three-phase boundary interface equipped with an efficient gas-circulation pathway, facilitating rapid mass transport of CO_2_. The GAME system can also be easily coupled with many other analytical techniques as exemplified by mass spectrometry (MS) and localized ultramicroelectrode (UME) voltammetry to enable real-time and in situ product characterization in the gas and liquid phases, respectively. The gaseous product distribution is explicitly and quantitatively elucidated with high time resolution (on the scale of seconds), allowing for the independent assessment of Tafel slope estimates for the hydrogen (159/168 mV decade^–1^), ethene (160/170 mV decade^–1^), and methane (96/100 mV decade^–1^) evolution reactions. Moreover, the UME is used to simultaneously measure the local pH shift during CO_2_RR and assess the production of liquid phase species including formate. A positive shift of 0.8 pH unit is observed at a current density of −11 mA cm^–2^ during the CO_2_RR.

## Introduction

Sustainable energy resources are being explored to alleviate global issues such as energy shortages and the greenhouse effect, thereby driving extensive research into electrocatalytic processes. The electrochemical CO_2_ reduction reaction (CO_2_RR) promoted by renewable energy to produce valuable chemicals and fuels has become a promising strategy for energy conversion, which can occur under ambient temperature and normal pressure conditions.^1^ In the meantime, it aids in mitigating the climate change problem by initiating a carbon-neutral cycle.^2^ However, it is a significant challenge to obtain the mechanistic information about the CO_2_RR as this is complicated by the multiple possible proton–electron transfer pathways and associated intermediates and is also accompanied by the competing hydrogen evolution reaction (HER).^3,4^ It is widely reported that the CO_2_RR performance (such as activity and selectivity) is strongly dependent on the nature and structure of catalysts and electrolytes.^5,6^ Among the catalysts used for CO_2_RR, Au predominantly leads to the production of CO,^7,8^ while Cu is the only metal that can generate hydrocarbon and alcohols with high efficiency.^9,10^

Another issue in relation to CO_2_RR research is the sluggish diffusion (0.0016 mm^2^ s^–1^) and low solubility (34 mM at 25 °C, 1 atm) of CO_2_ in aqueous solutions,^6,11−13^ hindering the evaluation of intrinsic kinetics and conversion efficiency. By enhancing mass transport through electrolyte advection, the rotating (ring) disk electrode (R(*R*)DE)^1,14^ and flow cell setups^15^ have been employed to investigate this reaction, leading to higher mass transport rates than under stagnant conditions. However, the gaseous reactant still needs to first dissolve before being utilized in the electrocatalytic reaction, and product gases are likely to be trapped and cause bubbles, disturbing electrolyte contact with the electrocatalyst. Moreover, they are not suitable for use of highly alkaline electrolytes (i.e., KOH) as a large amount of CO_2_ diffusing in the electrolyte would react with the electrolyte to form carbonate mixtures rather than participate in the electrocatalytic reactions.^16^ Instead, the development of gas diffusion electrodes (GDEs), which can create an efficient three-phase interface by reducing the diffusion path of the gases and allowing for the delivery of gases directly to the catalyst surface, has provided a remarkable scenario for the high-rate utilization and conversion of CO_2_.^17−19^

The product analysis of CO_2_RR, accounting for both the gas and liquid phases, plays a crucial role in understanding the reaction pathways. This usually requires a combination of many analytical techniques. Gas chromatography (GC) and mass spectrometry (MS) are the two main methods adopted to detect the gaseous and volatile products. The former is highly sensitive for quantification purposes, based on a periodic sampling protocol,^9,20^ while the latter reduces the detection time to the order of seconds, rendering it more suitable for real-time characterization in correlative techniques such as differential electrochemical mass spectrometry (DEMS)^21,22^ and online electrochemical mass spectrometry (OLEMS).^23^ The capability of online detection is highly useful, especially for the case when electrocatalyst deactivation readily happens.^24^ The liquid-phase products are normally detected by nuclear magnetic resonance (NMR), high-performance liquid chromatography (HPLC), and headspace GC,^25^ which are not capable of providing real-time responses, meaning that prolonged electrolysis has to be employed to ensure a measurable concentration of products.^9^ The RRDE technique offers a viable route to evaluate the liquid products,^20,26^ but besides low current densities, the detection is liable to be hindered by the generation and attachment of bubbles, although efforts toward tackling this issue have been made.^27,28^ Recently, intermediates and products have also been examined electrochemically by scanning electrochemical microscopy (SECM), a technique which is extremely sensitive and fast and particularly useful for the continuous detection of short lifetime and unstable species.^29,30^

Here, the gas accessible membrane electrode (GAME)^31^ system allowing for rapid mass transport has been implemented for the comparative study of CO_2_RR on Au and Cu–Au bimetallic electrodes. A well-defined three-phase interface can be readily formed at the 12 μm thick porous membrane electrode. The gas-circulation pathway of the GAME not only ensures a fast delivery of the CO_2_ reactant through the pores to the catalyst at the interface but also achieves rapid transport of the gaseous products to the MS connected in tandem with the GAME (GAME-MS) for real-time characterization. Meanwhile, an ultramicroelectrode (UME) has been positioned below the GAME to electrochemically diagnose the possible liquid products and probe the dynamic pH shift in the local environment during CO_2_RR. The GAME-MS-UME correlative platform enables a comprehensive study of CO_2_RR to be carried out with a high temporal and spatial resolution.

## Experimental Section

### GAME Preparation and Assembly

A custom-made GAME was used as the working electrode (WE) (Figure S1 in the Supporting Information), as described in our previous work,^31^ and is a development of an innovative approach to study electrocatalysis at the gas/liquid interface under high mass transport conditions.^32−34^ In brief, a 12 μm thick, porous polycarbonate track etch (PCTE, Sterlitech, *d* = 400 nm pores) membrane was first sputtered with a thin layer (∼100 nm) of Au to produce a conductive surface, named as Au/PCTE. After extensive cleaning in isopropanol and water in a Soxhlet extractor, the backside of Au/PCTE was brush-coated with a small amount of Teflon-AF (Chemours, dissolved in Fluorinert FC-40, Sigma-Aldrich) and left to dry in a vacuum oven at 70 °C.^35^ Then, it was mounted onto a polytetrafluoroethylene (PTFE) cylinder before being gently inserted into a PTFE tip. A flat surface can be obtained with an exposed electrode area of ∼0.35 cm^2^ sitting in the same plane as the end of the PTFE tip. Once electric contact is made to the Au/PCTE via a polyetheretherketone (PEEK) clip with Au wires (one for the WE and the other for the working sense (WS)) located in the middle of the cylindrical PEEK holder, the tip was tenderly twisted into the PEEK body. For the preparation of Cu–Au/PCTE electrode, the assembled GAME with an Au/PCTE electrode was immersed into a solution of 0.2 M CuSO_4_ and 1.5 M H_2_SO_4_ and polarized at −2 V for 20 s, while a Cu wire served as both the counter electrode (CE) and the reference electrode (RE). After in situ electrodeposition of Cu, the Cu–Au/PCTE electrode was dipped into DI water to remove the excess salts and then left to dry in air prior to the electrochemical measurements.

### CO_2_RR Performance Evaluation

The GAME was immersed in an electrochemical cell filled with a N_2_-degassed 0.5 M KHCO_3_ solution (pH ≈ 9.2), and the gas access to the electrode was manipulated from the top inlet by switching between N_2_ and CO_2_. A flow of 22 mL min^–1^ was regulated by a mass flow controller (Bronkhorst) during the electrocatalytic reactions. A Pt coil was flame-annealed to remove possible impurities prior to use, acting as the CE, while a leak-free Ag/AgCl served as the RE. Cyclic voltammetry (CV), chronoamperometry, and chronopotentiometry were performed. All the potentials reported in this work were iR-corrected (unless otherwise stated), in which the resistance R was determined using the high frequency real-axis intercept of electrochemical impedance spectroscopy from 100 kHz to 10 mHz with a 10 mV (root mean squared amplitude) sinusoidal modulation. They were further converted to the reversible hydrogen electrode (RHE) scale using
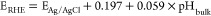
1Further corrections for local pH changes during operation will be described below.

### GAME-MS Measurements

The gas outlet of the GAME was connected to a quadrupole mass spectrometer (QGA, Hiden Analytical) using a PTFE tubing via a T-shape junction, allowing for the exit of excessive gas flow. An energy of 23 eV and ion current of 80 μA were adopted for mass spectrometry measurements. The calibration of the mass spectrometer was performed with a gas mixture of 1% H_2_, 1% CH_4_, 1% C_2_H_4_, and 1% C_2_H_6_ balanced with CO_2_ (Air Products).

### Electrochemical Characterization of the Liquid Phase by a Pt UME

To assess the changes in the liquid phase during the CO_2_RR on Au/PCTE, a UME was fabricated using a Pt wire (diameter 25 μm) sealed in a glass capillary and bent into a J-shape. The UME was then extensively polished to generate a flat and smooth end (RG ratio of ∼30). The electrode was rinsed with isopropanol and DI water, respectively, and then carefully translated close to the GAME, ensuring that the very end of the UME probe was right below the center of the exposed area of the GAME. The position of the UME in relation to the GAME was precisely controlled by a picomotor (Model 8321, Newport) mounted onto a three-axis translation stage, and the distance of the UME with respect to the GAME surface was estimated by the optical images captured by a purpose-built video microscope (PL-B776U camera, PixeLINK; VZM Zoom Imaging Lens, Edmunds Scientific) situated to the side of the electrochemical cell. A series of potential steps (−0.8 to −2 V vs Ag/AgCl, with a rest time of ∼310 s in between the steps) were applied to the GAME, while CVs were recorded simultaneously on the UME at a scan rate of 5 mV s^–1^.

### Physical Characterization

Scanning electron microscopy (SEM) images were obtained on a Zeiss Leo 1525 microscope. X-ray diffraction (XRD) analysis of the catalysts was conducted on a Bruker D2 PHASER diffractometer. In terms of ^1^H NMR measurements, the liquid species of CO_2_RR from Au/PCTE were characterized on a NMR spectrometer (Bruker AV400D) using a water suppression method. Typically, aliquots of 600 μL of solution after electrolysis at different potentials for 1 h were taken out and mixed with 50 μL of 50 mM phenol and 35 μL of 10 mM dimethyl sulfoxide (DMSO) prepared in D_2_O (used as internal standards) before measurements.

## Results and Discussion

### GAME-MS-UME Configuration and Catalyst Characterization

**Figure 1:**
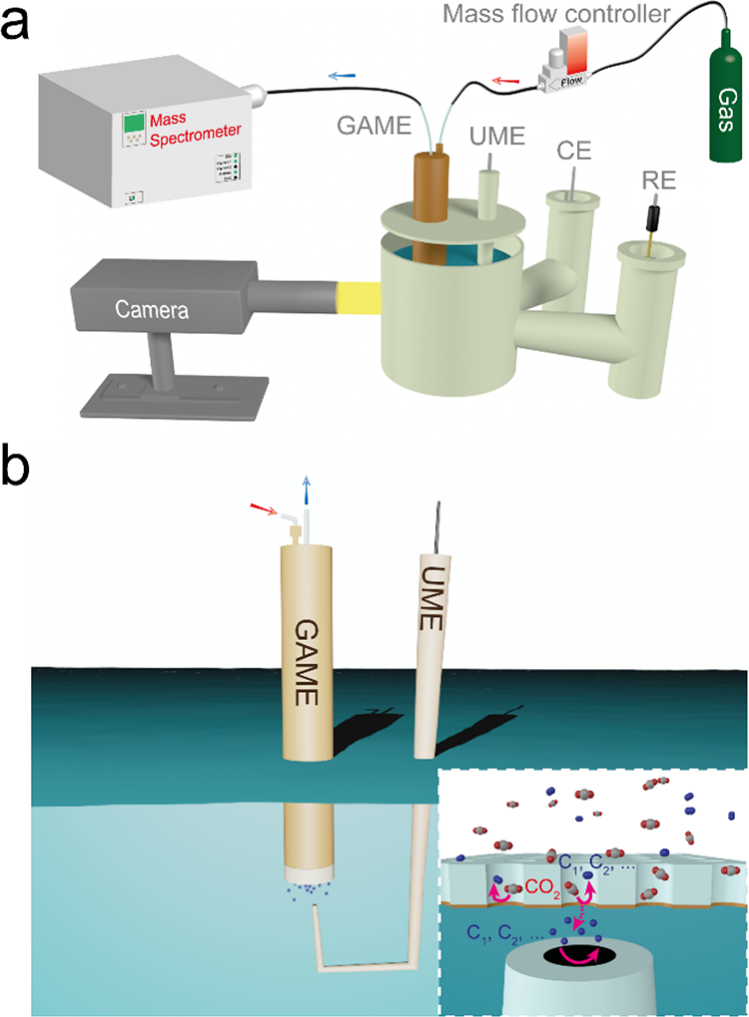
Schematic illustration for (a) the correlative configuration for CO_2_RR measurements and (b) the zoom-in region of the GAME-UME, with the inset highlighting the reaction interface and regime in the local environment. The gas inflow and outflow are indicated by red and blue arrows, while the reaction pathways in the local environment are shown with pink arrows.

The experimental setup for CO_2_RR study is shown in Figure 1. The carrier gas (either N_2_ or CO_2_) circulates past the GAME under a rate regulated by a mass flow controller, while any gaseous products generated by CO_2_RR are simultaneously transported out of the GAME and sent to the coupled MS for online analysis. In some cases, a Pt UME is positioned coaxial to the GAME to probe the liquid products diffusing from the surface (Figure 1 and Figure S2a of the Supporting Information). The separation between the UME and the GAME is set (tens to hundreds of μm) using an optical video microscope. The gas exchange regime within the GAME is further illustrated in Figure S2b. The incoming gas is introduced to the chamber (∼0.64 cm^3^ volume) of the GAME through the inlet and then diffuses through the hydrophobic pores of the membrane electrode, establishing a highly efficient triple-phase reaction interface. Meanwhile, the volatile and gaseous products can mostly ex-solve into the hydrophobic pores, return to the chamber, and subsequently exit from the chamber with the remaining carrier gas via the outlet. Of note, the GAME substrate (ca. 12 μm thick) is about 20× thinner than the typical gas diffusion electrodes used in fuel cells and CO_2_ electrolyzers (∼250 μm), and hence, the reactant/product transport is significantly enhanced.^31,35^ Therefore, this configuration facilitates high reaction rates, rapid gas feed and collection, and real-time product distribution analysis (gases by the coupled MS, and liquids by the adjacent UME, vide infra).

**Figure 2:**
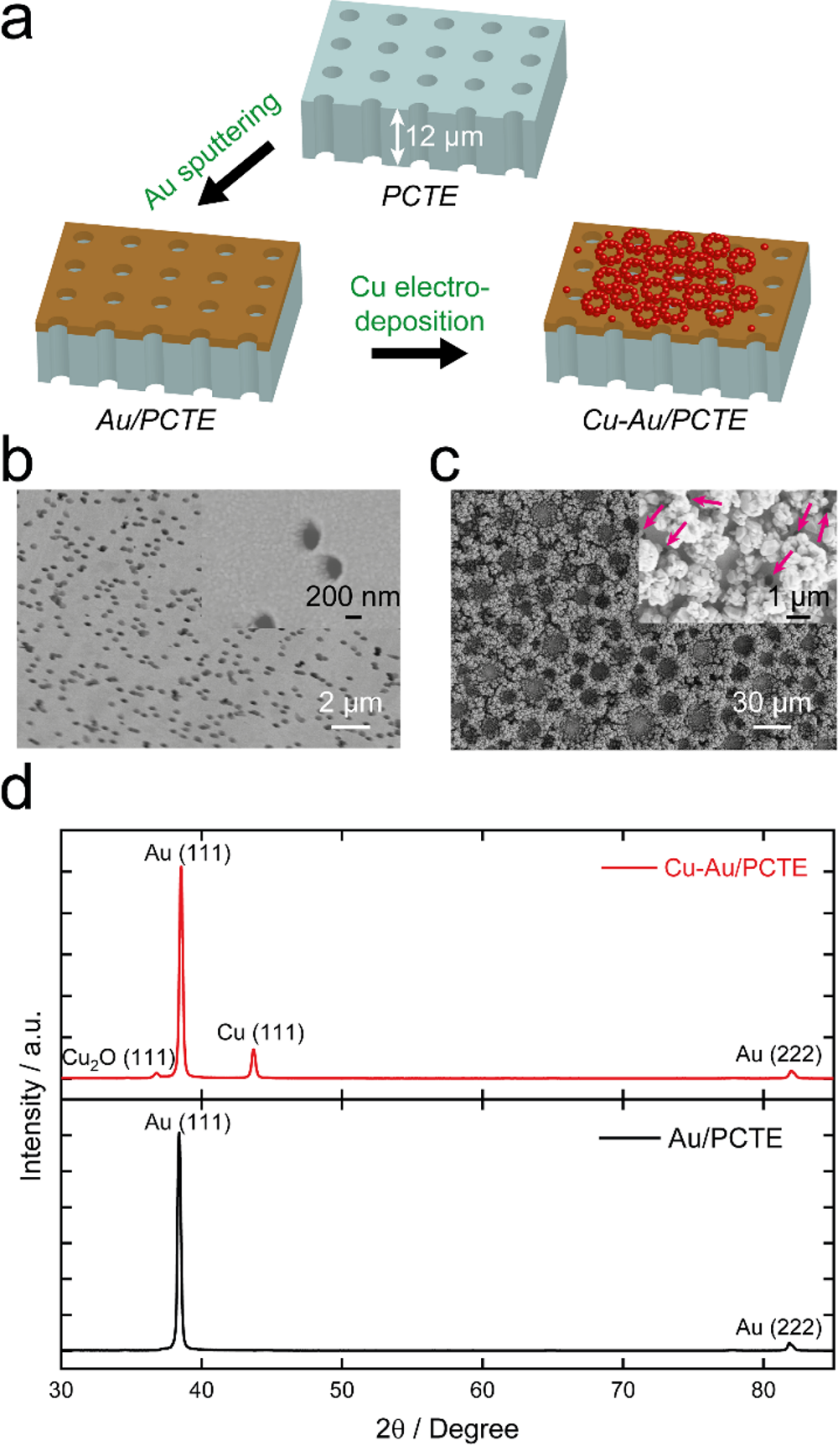
(a) Schematic of the sample preparation process (not to scale). (b, c) SEM images of the Au/PCTE and Cu–Au/PCTE, respectively. High-resolution images are shown in the insets. The positions of open pores after Cu electrodeposition are indicated with pink arrows. (d) XRD patterns of Au/PCTE and Cu–Au/PCTE electrodes.

In this work, we have examined the performance of two catalysts loaded onto the PCTE with significantly different activity and selectivity toward the CO_2_RR, i.e., Au and Cu–Au (Figure 2a). It is reported that the Au catalysts mainly lead to the formation of H_2_ and CO, while Cu is the only metal that facilitates the generation of hydrocarbons and alcohols.^6,36^ Bimetals can alter the adsorption energies for reactants and intermediates.^37,38^ Here, the Au layer sputtered on PCTE previously adopted as the conductive substrate for the floating electrode technique is directly used to provide the Au catalyst herein. As shown in Figure 2b and Figure S3, the Au/PCTE sample displays a notably reflective surface. The sputtered Au forms a continuous layer (∼100 nm thick) on the PCTE and also possibly coats the inner surface of the pores^39^ yet still leaving the pores open for gas transportation (as observed in the SEM image; Figure 2b). The as-prepared Au layer can play multiple roles on the GAME: serve as a conductive film on the PCTE membrane to collect electrochemical current, act as a uniform and flat catalyst for electrochemical reactions (e.g., CO_2_RR in this work), and provide a templated substrate on which other structures can be introduced. It is worth mentioning that the sputtering strategy for electrode preparation enables a wide variety of materials to be potentially used as the WE subject to the research purposes. We have previously used the same electrode structure to study other electrochemical reactions such as oxygen evolution/reduction and hydrogen evolution/reduction. A comparison between the electrode structure used in the GAME and that typical of the gas diffusion electrodes used in fuel cells (and by inference, CO_2_ electrolyzers) is provided in a paper by one of us.^35^

Cu was subsequently electrodeposited onto the same sample of Au/PCTE (at −2 V for 20 s) to generate a bimetallic catalyst Cu–Au, ensuring a direct comparison of the electrocatalytic behavior with Au. As illustrated in Figure 2c and Figure S3b, after deposition of Cu on the Au/PCTE GAME, the electrode surface becomes pink and some nanofoam structures (although the extent and coverage are highly dependent on the electrodeposition time) are observed as a result of the uneven electrolyte accessibility templated by H_2_ bubbles vigorously generated at very negative potentials.^40^ Since the majority of Au is sputtered on the solid part of the PCTE membrane (accounting for 87.4% of the overall area)^41^ rather than into the pores, Cu electrodeposition would mainly occur over the solid portion. Therefore, there are still a large number of open pores present on Cu–Au/PCTE, allowing for the efficient gas transport through the PCTE substrate (inset of Figure 2c pointing out the pores). In both cases mentioned above, when the gas is supplied to the GAME, a consistent three-phase (gas–solution–catalyst) interface is generated in an electrolyte and the structure does not “flood”.^35^ The scenario that the catalyst layer is situated adjacent to the interface of CO_2_ gas and electrolyte allows for the continuous feed of gaseous CO_2_ to the reaction interface even under alkaline conditions, where significant consumption of CO_2_ by the electrolyte would be expected in conventional setups.^42^ Moreover, the open pores of the GAME enables the resulting gaseous and volatile products to migrate into the GAME chamber before being sampled for online analysis (vide infra). XRD patterns of the two electrodes in Figure 2d show that on Au/PCTE, there are mainly two phases: Au (111) and Au (222) crystal facets,^43^ and after Cu electrodeposition, Cu(111) and Cu_2_O(111) are introduced.^44^

### Electrochemical Response in the Presence of CO_2_

**Figure 3:**
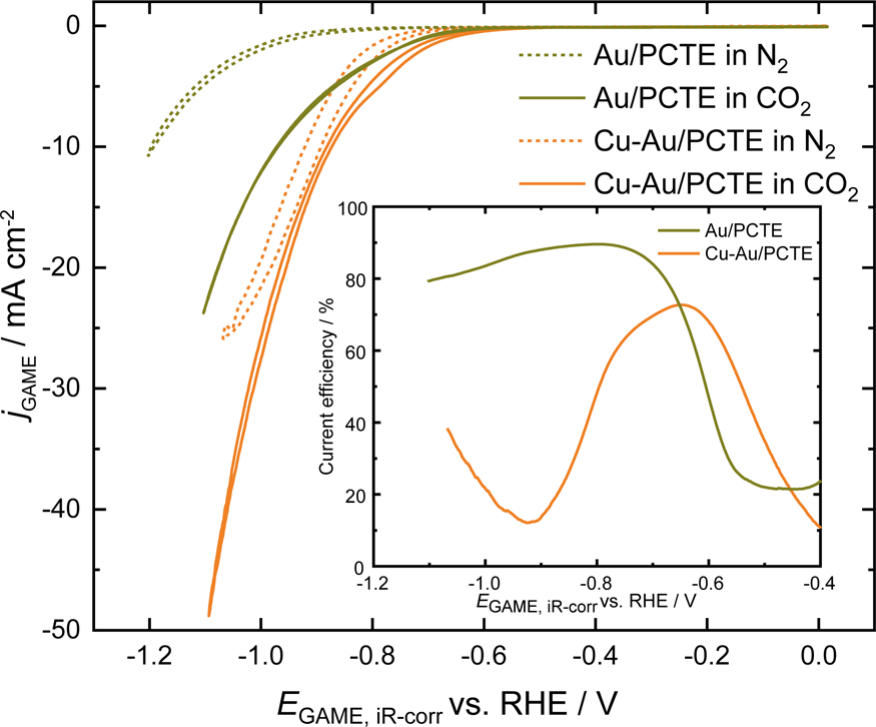
CO_2_RR cyclic voltammograms of Au/PCTE and Cu–Au/PCTE electrodes recorded in a solution of 0.5 M KHCO_3_ under N_2_ atmosphere and CO_2_ atmosphere, respectively. Scan rate: 5 mV s^–1^. The inset shows the current efficiency of the CO_2_RR on Au/PCTE and Cu–Au/PCTE, respectively.

To evaluate the electrochemical behaviors of these two electrodes toward the CO_2_RR, CV was first carried out using a three-electrode system. It is noteworthy that the bulk solution of 0.5 M KHCO_3_ was saturated with N_2_ (pH 9.2) prior to the measurements, and this was then maintained in an airtight cell for the whole experiment. This strategy can have two benefits: first, a higher pH achieved by N_2_ saturation (than that with CO_2_ saturation) can facilitate better conversion during the CO_2_RR;^42,45^ second, the capability of efficient gas delivery and exchange associated with the GAME system can avoid the extensive use of gases for continuously sparging the solution over the course of measurements commonly adopted in conventional setups. When the Au/PCTE is exposed to N_2_ atmosphere (Figure 3), the reduction current increases slowly with the decreasing potential, which is purely ascribed to the HER process. As CO_2_ is introduced into the headspace, an onset potential (the potential at which 1% of the maximum value is recorded) of −0.62 V is observed (compared to −0.65 V for the sample under N_2_) (see the Supporting Information), and the cathodic current rises dramatically due to the beginning of the CO_2_RR. At −1.07 V, a geometric current density of −19.3 mA cm^–2^ (geometric area: 0.35 cm^2^, with a roughness factor of ∼4 (Supporting Information, Figure S4) is recorded, which is much higher than those reported in the literature.^46^ The enhanced current should be composed of the responses from both the HER and CO_2_RR. To this end, the Faradaic current of CO_2_RR can be estimated by subtracting the HER contribution on its own (dashed line) from the overall response. The current efficiency for CO_2_RR on Au/PCTE reaches a maximum value of ∼90% and decreases with the increase of overpotentials due to the enhanced HER (inset of Figure 3; also see the Supporting Information).^47,48^ Note that the current density can be improved further by optimizing the structural design of the gold electrodes (as for the current electrodes, only the Au sputtered near the pores are expected to have access to the inlet CO_2_ gas) and by increasing the feed rate of CO_2_.^49,50^

Upon the electrodeposition of Cu onto Au/PCTE, the CV under an N_2_ atmosphere demonstrates a much steeper increase in electrochemical current than that for the Au/PCTE after the onset potential of −0.69 V. When the headspace gas is switched to CO_2_, slightly higher currents are observed until −0.9 V, after which, with the increase of the cathodic potential, the current grows dramatically (Figure 3). A current density of ∼42.4 mA cm^–2^ is observed at −1.07 V on Cu–Au/PCTE in the presence of CO_2_ in comparison to 26 mA cm^–2^ under N_2_. The currency efficiency for CO_2_RR on Cu–Au/PCTE shows a different pattern from that on Au/PCTE. It achieves a maximum of 73% at −0.65 V followed by a decrease to a minimum value of 12% at −0.92 V and, subsequently, another increase beyond this peak. The trend matches reasonably well with the literature.^47^ Note that during the CV measurements presented here, it is found that the GAME is immune to gas bubble formation due to the highly efficient gas transport capability—this is a significant advantage to what is commonly seen in the R(*R*)DE techniques.^28^ The discrepancy in the electrochemical performances of the two electrodes indicates that the CO_2_RR behaviors can be tuned by Cu electrodeposition onto the electrode. Interestingly, there is little hysteresis in the forward and reverse scans, indicating that the conditions at the electrocatalyst surface are quickly adjusted and the response is not significantly affected by the history of the electrode. This also suggests that any local pH effects are instantly established within the timescale of the experiment.

### Electrochemical–Mass Spectrometric Response of the Gas-Phase Products

Next, we employed the GAME-MS to elucidate the relationship between the activity and selectivity of CO_2_RR on the two electrodes from the perspective of product distribution. In the GAME setup, the catalysts (both Au and Cu–Au) are directly deposited onto the porous PCTE membrane in the vicinity of the pores, enabling the reactant gas, CO_2_, to travel only a short distance before being utilized by CO_2_RR.^31^ Moreover, the electrocatalytic reaction sites and gas sampling interface are colocated, but product detection does not impact product generation, allowing for the simultaneous collection of the gaseous product species diffusing into the GAME for further evaluation. Therefore, the delay time for the gas-phase products to be detected after generation can be minimized.^15^ In the present GAME-MS configuration, the characteristic delay time is measured to be ∼7–8 s (Supporting Information), shorter than many conventional vacuum-based OLEMS setups.^51^ The fast response characteristic of this technique enables us to monitor the gas-phase product distribution of CO_2_RR in real time. Compared with the accumulative product analysis approach by GC, the online product characterization by the GAME-MS can enable the reaction pathways, dynamics, and kinetics of CO_2_RR to be potentially deciphered. In this study, the H_2_ (*m*/*z* = 2), CH_4_ (*m*/*z* = 15), and C_2_H_4_ (*m*/*z* = 26) molecules generated from CO_2_RR are simultaneously detected. It is noteworthy that in the current work and many others, the CO product is genuinely difficult to probe since the fragmentation of carrier gas (i.e., the flowing CO_2_) can lead to considerable background signals at *m*/*z* = 28,^52−54^ and the overlap hampers the deconvolution of CO. Although the method of subtracting the estimated contribution of CO_2_ from the overall *m*/*z* = 28 response has been applied in some studies, large errors can be potentially introduced.^55^ Instead, the optimization of the spectrometer by tuning the electron energy to modulate the fragmentation may offer a more practical and accurate approach. The detailed exploration will be the focus of a future work.

Chronoamperometry was first performed on the two different electrodes, while the MS signals (i.e., molar flow rate) were recorded. In the case of Au/PCTE, there is only H_2_ formation observed during the reaction either under N_2_ or CO_2_ (Supporting Information) since CO is not considered for the time being. In contrast, the Cu–Au/PCTE sample demonstrates the formation of H_2_, C_2_H_4_, and CH_4_ under a CO_2_ atmosphere (Supporting Information), and the molar flow rates correspond to the electrochemical potentials (and the resulting currents) very well. The onset potentials are shown to be in the order of H_2_ > C_2_H_4_ > CH_4_, in line with the literature using Cu electrodes.^36,56^ Moreover, as the potential is stepped from −0.83 to −1.1 V (iR-corrected potentials), the mass flow fraction (MFF) of H_2_ is decreased from 98.6 to 88.9%, while the MFF of C_2_H_4_ is increased from 1.4 to 10.6%. However, the MFF of CH_4_ only shows a small value of 0.5% even at −1.1 V. These results indicate that the selectivity of CO_2_RR on Cu–Au/PCTE is highly potential-dependent and also highlight the good collection efficiency of the GAME. The CO_2_RR performance on Cu–Au/PCTE is further investigated with chronopotentiometry, and the MS signal promptly follows the stepwise change in Faradaic current. Only H_2_ evolution is observed at a very low current of −0.5 mA (−1.43 mA cm^–2^), while C_2_H_4_ is generated from −2 mA (−5.71 mA cm^–2^) and CH_4_ can only be detected with a current of −8 mA (−22.86 mA cm^–2^). Under an applied current of −14 mA (−40 mA cm^–2^), the averaged MFF values for the H_2_, C_2_H_4_, and CH_4_ are approximately 88, 10, and 2%, respectively. Therefore, a fast evaluation of the MFF can be obtained in response to the sequential steps.

**Figure 4:**
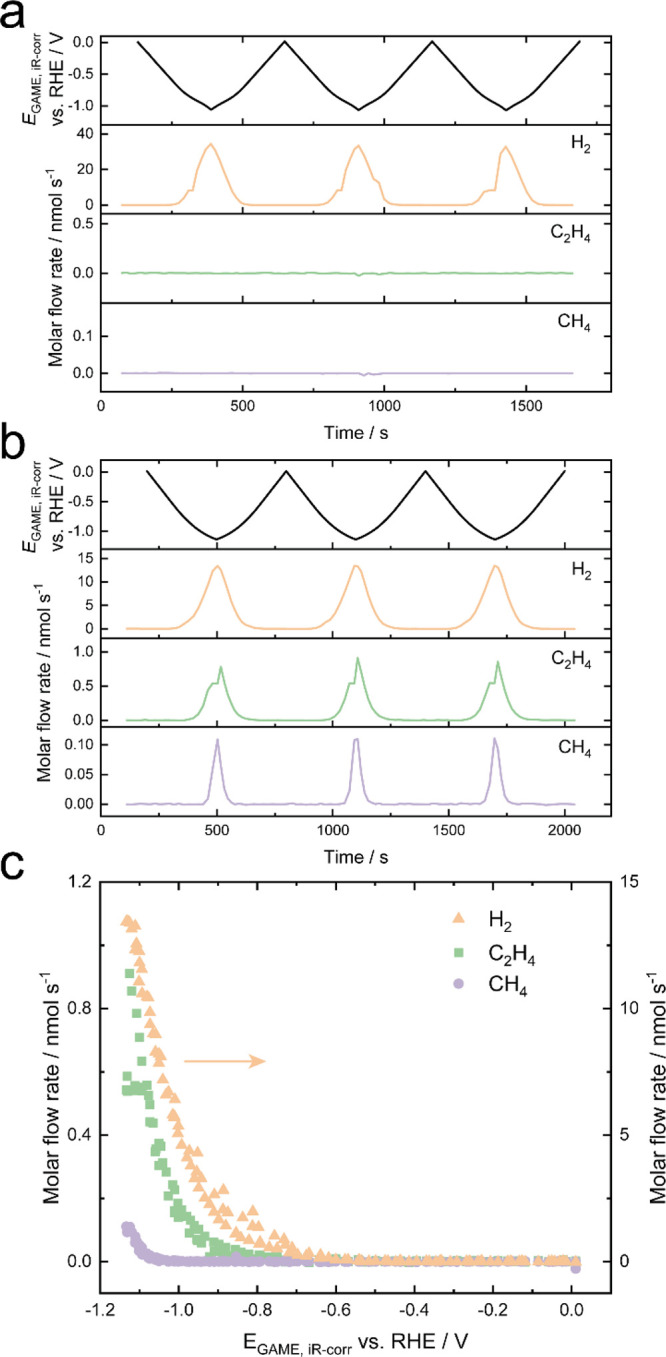
Molar flow rates for H_2_ (*m*/*z* = 2), C_2_H_4_ (*m*/*z* = 26) and CH_4_ (*m*/*z* = 15) during CV scans recorded on Cu–Au/PCTE at 5 mV s^–1^ in N_2_-saturated 0.5 M KHCO_3_ solution under (a) N_2_ and (b) CO_2_ conditions, respectively. (c) MSCVs plotted with data shown in (b) to provide a iR- and time-corrected voltammogram of the instantaneous rate of product generation.

However, the aforementioned experiments are performed when the reactions proceed under a steady state (constant potential or current), and this strategy has a somewhat limited ability to scrutinize the potential-dependent nature of the CO_2_RR process since the resolution is determined by the step size of the potentials/currents. To this end, MS responses were also simultaneously recorded over the CVs of Cu–Au/PCTE to elucidate the dynamic formation of CO_2_RR products. As shown in Figure 4a, there is only H_2_ detected during the CV performed at 5 mV s^–1^ under N_2_ atmosphere. However, when the headspace gas is altered to CO_2_ (Figure 4b), C_2_H_4_ and CH_4_ are also produced. The evolution of C_2_H_4_ and CH_4_ is affected by the potentials, and the molar flow rates of both species are improved with the scan to more negative potentials. These MS results show excellent reproducibility over successive voltammograms. Furthermore, it should be noteworthy that no elongated current tail is observed for the transient MS signals, as well as the steady-state MS responses (Supporting Information), indicative of a fast mass transport without bubble accumulation.^57,58^ In Figure 4c, the instantaneous evolution of products is further illustrated as a function of iR-corrected potential (MSCV; based on the data in Figure 4b). This again shows that there is little hysteresis in the response, and the forward and reverse scans overlay one another. This confirms that the system does not suffer from the artifacts introduced through low-performance electrodes with poor mass transport.

**Figure 5:**
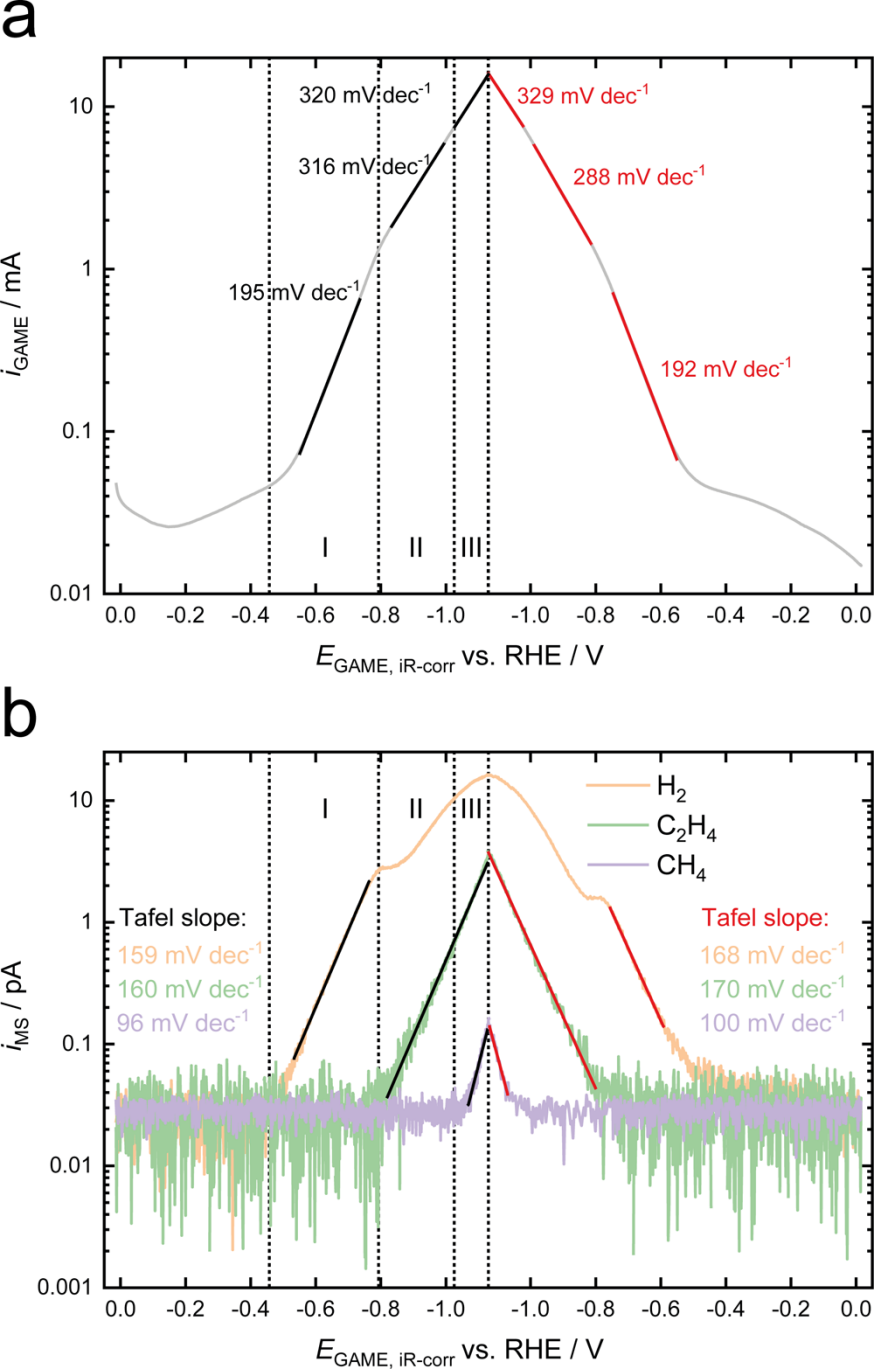
(a) Unfolded Faradaic current–potential profile from a CO_2_RR CV of Cu–Au/PCTE recorded in 0.5 M KHCO_3_ at a scan rate of 1 mV s^–1^. (b) Corresponding mass ion current responses for H_2_ (*m*/*z* = 2), C_2_H_4_ (*m*/*z* = 26), and CH_4_ (*m*/*z* = 15), respectively, during the CV scan shown in (a). Estimated Tafel slopes are indicated.

In order to assess the kinetic properties, the CO_2_RR CVs of Cu–Au/PCTE were further recorded at 1 mV s^–1^, using the GAME-MS setup. As shown in Figure 5a, the Faradaic current varies exponentially with the potential in three zones during the negative-going sweep, with Tafel slopes of 195, 316, and 320 mV dec^–1^, respectively. It is noteworthy that in the positive-going scan, comparable Tafel slope values are obtained, i.e., 192, 288, and 329 mV dec^–1^, respectively, indicating that the reaction is in a relatively steady state for the same potential regions between the forward and back scans. Correspondingly, from the unfolded MSCV data displayed in Figure 5b, the onset potentials and the mass ion current (*i*_MS_) magnitudes of the gaseous species follow the trend H_2_ > C_2_H_4_ > CH_4_. The onset potentials and Tafel slopes obtained from different techniques are summarized in Table 1. It can be seen that these data from the CV are in good accordance with the results from chronoamperometry and chronopotentiometry. The discrepancy in onset potentials and Tafel slopes can be due to the application of different experimental protocols (stepwise vs continuous) and the variation of potential regions for data analysis, respectively. Overall, the MSCV method provides a more precise way of evaluating the onset potentials for the products and the Tafel slopes since a wide range of dynamically changing potentials can be investigated. Moreover, the mass ion currents also show a Tafel-like potential dependence, with domains that match those of the *I**–**E* curve. Likewise, they can be divided in three zones for the same potential ranges as Figure 5a in the negative-going scan. In Zone I, only H_2_ is produced and a linear relation between the *i*_MS_ in a logarithmic scale and the potential is seen, leading to a well-defined estimated Tafel slope of 159 mV dec^–1^ over a range of two orders of magnitude in *i*_MS._ As the *i*_MS_ is exclusively associated with the production of hydrogen, this Tafel slope is the same as expected for the electrochemical partial current for the hydrogen evolution reaction, leading to gaseous hydrogen. Hence, the *i*_MS_ currents can be used to determine the Tafel slopes of the appropriate reactions provided that there are no other (non-gaseous) sinks for the produced species (i.e., under the condition where all hydrogen goes into the gas stream and is not consumed in any following processes). Next, after ∼340 s (equivalent to a potential difference of 0.34 V), C_2_H_4_ starts to appear in Zone II and the *i*_MS_ varies exponentially with a Tafel slope of 160 mV dec^–1^, accompanied by a shoulder peak in the *i*_MS_ of H_2_, after which the production of H_2_ increases at a much slower rate. Once the overpotential is increased further by −0.23 V (ca. 230 s) to Zone III, the *i*_MS_ for CH_4_ commences, showing an estimated Tafel slope of 96 mV dec^–1^, while the rate of H_2_ increase is further inhibited, and the rate of C_2_H_4_ increase is unchanged. In the following positive-going scan, similar slope values are seen for the three products in three respective zones, with a summary of the average values of the estimated Tafel slopes provided in Table 2. Note that the difference in the slopes obtained from the Faradaic and mass ion currents lies in the fact that the former is contributed by all the products (including any extra ones which dissolve in the electrolyte), while the latter is exclusively from a specific species.^59^ However, the results presented here highlight that the kinetic patterns of product evolution are crucial to explain the Tafel slope changes observed for the *I***–***E* curve over different potential regions and in opposite scan directions. The significant deviation from Tafel behavior seen for the hydrogen signal in Figure 5b once ethene production commences (and further seen when methane production starts) is hardly surprising as it is transitioning from a regime where all of the H_ads_ produced on the electrode forms H_2_ to one in which the H_ads_ can either form H_2_ or be consumed in other reactions, leading to ethene and methane.

**Table 1:** Summary of the Onset Potentials for the Production of Different Species as Determined from MS Gas Analysis

	onset potential (V vs RHE)		
technique	H_2_	C_2_H_4_	CH_4_
chronoamperometry	–0.66	–0.83	–1.10
chronopotentiometry	–0.72	–0.87	–1.06
cyclic voltammetry	–0.59	–0.81	–1.04

**Table 2:** Average of Forward and Reverse Estimated Tafel Slopes Based on Electrochemical Current and MS Current for Different Species Derived from Cyclic Voltammograms in Each of the Three Regions Demarcated in Figure 5^a^

	estimated Tafel slope (mV dec^–1^)			
		mass spectrometric response		
potential region (V vs RHE)	electrochemical current	H_2_	C_2_H_4_	CH_4_
Zone I (−0.46 to −0.79)	194	164	NS	NS
Zone II (−0.79 to −1.03)	302	ND	165	NS
Zone III (−1.03 to −1.13)	325	ND	165	98

a ND: not developed, curved response indicating no defined Tafel slope; NS: no signal, potential too low to generate measurable amounts of species.

### Correction of Local pH Shift Using Ultramicroelectrode (UME) Response

So far, within this work, the interpretation of gaseous species for CO_2_RR has been extensively explored, while the evaluation of products in the electrolyte remains unclear. Given the complex sampling procedures and long analysis time associated with the conventional techniques, such as HPLC and NMR, it is vital to develop efficient analytical tools to enable the online and in situ inspection of local reaction environment. Due to the merits of high sensitivity and fast response, localized electrochemistry using UMEs provides promising approaches for continuous real-time measurements of liquid species. In many ways, the arrangement of a UME adjacent to the GAME is similar to the use of a ring electrode in the RRDE technique. Therefore, the correlative configuration of a UME and online MS coupled with the GAME, i.e., GAME-MS-UME, facilitates the simultaneous diagnosis of both the liquid and gas products of CO_2_RR.

In this work, the CO_2_RR on the Au/PCTE electrode is used as a model system to demonstrate the capability of coupled GAME-MS-UME given its relatively simple reaction routes (and therefore product species) compared with other metals. A Pt UME, which has a diameter of 25 μm, can be positioned adjacent to the GAME (≤100 μm away; see the Supporting Information). The potential at the UME is cycled, while a series of constant potential steps are applied to the GAME purged with N_2_ or CO_2_. During the CO_2_RR of Au/PCTE at the GAME (equivalent to the generator in the SECM technique), the concentration of product species in the liquid can be replenished constantly across the gap, ensuring a steady mass transport profile at the UME (analogous to the collector in SECM), which can then act as a powerful probe to electrochemically detect them immediately after generation.

**Figure 6:**
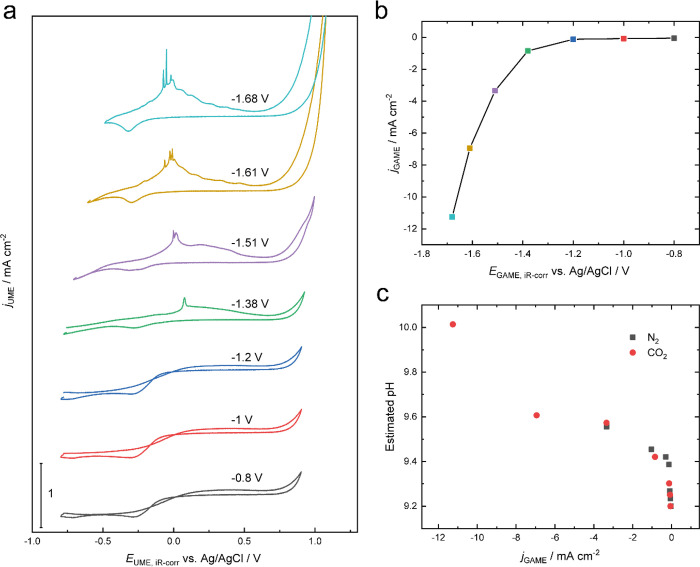
(a) UME CVs recorded at 5 mV s^–1^ when the GAME is held at different potentials. (b) Averaged current densities on the GAME under different potential steps. (c) Estimated pH of the electrolyte in the vicinity to the GAME as a function of GAME current density under an N_2_ or CO_2_ atmosphere.

As expected, the GAME demonstrates higher Faradaic currents in CO_2_ than in N_2_ (see the Supporting Information), and the MS results show that H_2_ is the only detectable gaseous product for both cases (Figure S8). However, there are some distinct features on the UME CVs recorded when the GAME is supplied by N_2_ or CO_2_. Note that given that the electrochemical measurements were carried out using a four-electrode system, the Ohmic drop on the GAME electrode also affects the potential at the UME, and the cross-talk effect between the two working electrodes needs to be carefully considered (Supporting Information).^60−63^ Under N_2_, there is only a pronounced peak observed in the cathodic scan of the UME CVs corresponding to the reduction of Pt oxide (a proton-consuming process; see the Supporting Information). Next, when the headspace in the GAME is fed with CO_2_, an oxidation wave on the UME CV is seen. This peak shifts negatively, and the magnitude is improved with the decrease of the potential on the GAME (Figure 6a and Figure S17). This behavior is very reproducible over a range of measurements (Figure S18) and will be discussed in detail later. Meanwhile, the current density on the GAME is increased significantly below −1.4 V vs Ag/AgCl (Figure 6b), and these values measured at constant potentials match reasonably well with those observed during voltammetry on the GAME, albeit on different samples (see Supporting Information, Figure S19).

Another characteristic of the UME CVs is that the reduction peak in the presence of CO_2_ shifts to more cathodic potentials, while for the CVs recorded under N_2_, this is only seen at very negative potentials and with a much smaller magnitude. This behavior is a reflection of the increase of the local pH due to the release of hydroxide ions during CO_2_RR and HER, which can further possibly influence the product selectivity.^56,64,65^ Therefore, we have used the voltammetric response of Pt oxide reduction on the UME as a sensor for the local pH variation.^66^ The potential difference is then converted to pH values using the Nernst equation (see the Supporting Information). By this means, the extent of pH shift during electrochemical reactions can be in situ assessed without the extra effort of electrode modification. The relationship between the pH variation (from the original value of 9.2 for the bulk N_2_-saturated solution) and the current densities of the GAME is depicted in Figure 6c, analogous to the behavior reported previously.^67,68^ Two correlation regimes are seen, regardless of the type of gas fed to the GAME: at small current densities (>−0.3 mA cm^–2^), the local pH changes fast; at high current densities (<−0.3 mA cm^–2^), the pH variation slows down with the increasing current density. Under the highest current density studied herein (−11.26 mA cm^–2^), the local pH increases by 0.8 unit. Note that this value is distance-dependent since a gradient of pH is established around the GAME, which can be fully characterized by varying the separation between the GAME and the UME.

### pH-Corrected Response of the Solution-Phase Products at the UME

**Figure 7:**
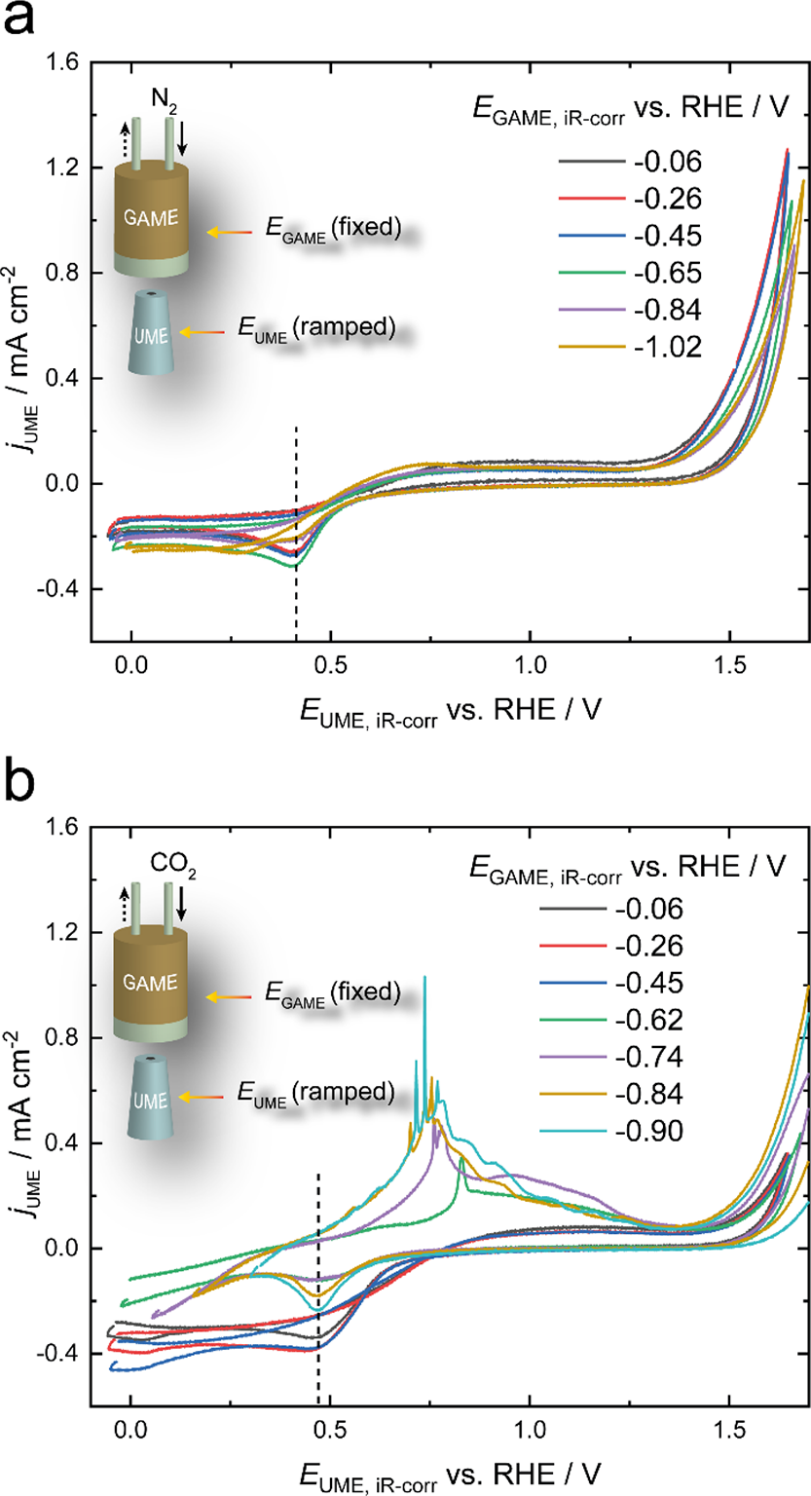
Cyclic voltammograms of the Pt UME (*d* = 25 μm) obtained in N_2_-saturated 0.1 M KHCO_3_ when the Au/PCTE GAME is supplied with (a) N_2_ and (b) CO_2_. Scan rate: 5 mV s^–1^. Insets show the schematics for the setup where a series of constant potentials are applied to the GAME, while CVs are recorded on the Pt UME.

The potentials of the UME CVs were further corrected with respect to the RHE scale to take into account the pH shift effect induced by the CO_2_RR on the GAME based on the discussion above. As shown in Figure 7, the reduction peak for Pt oxide is now aligned for all the voltammograms. During the electrolysis of Au/PCTE under N_2_, CVs almost overlap in the range of 0–1.0 V and no oxidation peak is unambiguously observed. In contrast, when the headspace in the GAME is switched to the CO_2_ atmosphere, an oxidation peak is seen on the UME CVs when the potential on the GAME is kept at less than or equal to −0.62 V vs RHE (−1.38 V vs Ag/AgCl). The anodic wave on the UME shifts negatively and increases in intensity with the further decrease of the potential of the GAME. In some cases, there is another oxidation peak observed in the back (negative-going) scan (Supporting Information). These electrochemical responses are likely to be predominantly ascribed to the oxidation of CO and HCOO^–^ products (see the NMR analysis of solution in the Supporting Information), analogous to the performance reported elsewhere.^26,30,69^ At −0.62 V, there is a sharp peak ascribed to the CO electro-oxidation on platinum. Note that in our case, most of the resulting CO would diffuse into the gas channels of the porous electrode and be collected in the gas phase, less likely to form bubbles to interfere with the local electrochemical measurements, in contrast to the R(*R*)DE and SECM techniques.^28^ However, at lower potentials (≤−0.74 V on the GAME), the peak shifts to lower potentials with an enhancement of the currents, indicative of formate oxidation. Some variance seen across repetitive measurements is possibly caused by several factors, such as the concentrations of products, the separation between the GAME and the UME, and the dynamic pH (dependent on the extent of the electrocatalytic reaction; see the Supporting Information). Note that the current detection of liquid products has not yet been directly correlated to the evolution of gaseous products interrogated by the GAME-MS, which will be explicitly investigated in the future.

## Conclusions

In this study, a correlative platform of GAME-MS-UME has been developed to extensively investigate the CO_2_RR process. This configuration enables fast gas distribution and product collection to be simultaneously achieved while serving as a powerful tool for real-time and in situ characterization. From the synchronized electrochemical–spectrometric results, the evolution of diverse CO_2_RR products (H_2_, C_2_H_4_, and CH_4_) is elucidated, providing insights about the onset potentials for individual products and the Tafel slopes for the overall reaction. It should also be highlighted that the estimated Tafel slopes for individual products can be obtained from the MSCVs enabled by the GAME-MS, which are critical to the interpretation of electrokinetic behavior. Such information will be useful in revealing the possible reaction pathways leading to these products. Moreover, the scenario of using the UME to evaluate the pH shift and the liquid products over the course of the CO_2_RR further adds to the experimental approaches to investigate the pH effect, which is yet mostly assessed by theoretical means. Therefore, the high-resolution characterization presented in this work offers paramount perspectives for unraveling the mystery of complex electrochemical processes, making it superior to many other analytical methods. In future, this hyphenated technique can be further improved to complete the full-spectrum product analysis, including CO, and extended to study many more catalysts and reactions. It also opens up prospects for the continuous production of targeted chemicals in combination with flow cells.

## Supporting Information Available

Schematics for the GAME-MS-UME; optical images of the prepared samples; determination of onset potentials and current efficiency; estimation of ECSA of Au/PCTE; response time assessment of the GAME-MS; chronoamperometry and chronopotentiometry measurements and MS responses for CO_2_RR; electrical cross-talk effect; characterization of the Pt UME and UME CVs; estimation of pH during CO_2_RR; NMR spectra for the liquid products (PDF)

## Notes

The data used for the production of the figures in this work are available to download at DOI:10.5281/zenodo.6526650.
