# Supplementary material for: Real-Time In Situ Monitoring of CO2 Electroreduction in the Liquid and Gas Phases by Coupled Mass Spectrometry and Localized Electrochemistry
Source: ACS Catal. 2022 May 10;12(10):6180–90. doi: 10.1021/acscatal.2c00609 (PMC9127967; doi:10.1021/acscatal.2c00609)
Supplement: Supplementary file 1 — cs2c00609_si_001.pdf [file cs2c00609_si_001.pdf]

Supporting Information

Real-Time In Situ Monitoring of CO_2 Electroreduction in the Liquid and Gas Phases by Coupled Mass Spectrometry and Localized Electrochemistry

Guohui Zhang,† Youxin Cui, Anthony Kucernak*

Department of Chemistry, Imperial College London, London SW7 2AZ, United Kingdom

*Email: anthony@imperial.ac.uk

†Current Address: State Key Laboratory of Catalysis, Dalian Institute of Chemical Physics, Chinese Academy of Sciences, Dalian 116023, P.R. China

Contents

S1| The structure of the GAME and associated positioning of the UME probe S-2

S2| Onset potential and current efficiency determination S-4

S3| Estimation of ECSA of Au/PCTE S-5

S4| The response time of mass spectrometric signals S-6

S5| Chronoamperometry and MS responses of Au/PCTE S-9

S6| Chronoamperometry and chronopotentiometry measurements for the CO_2RR on Cu-Au/PCTE S-11

S7| Electrochemical characterisation of the UME probe S-16

S8| Electrical cross-talk effect in the current configuration S-18

S9| Pt UME responses during chronoamperometry S-19

S10| Estimation of pH during the electrolysis under constant potential steps on the GAME S-21

S11| UME CV during chronoamperometric measurements on the GAME S-22

S12| NMR spectra of solutions after electrolysis S-23

References S-24

S1| The structure of the GAME and associated positioning of the UME probe

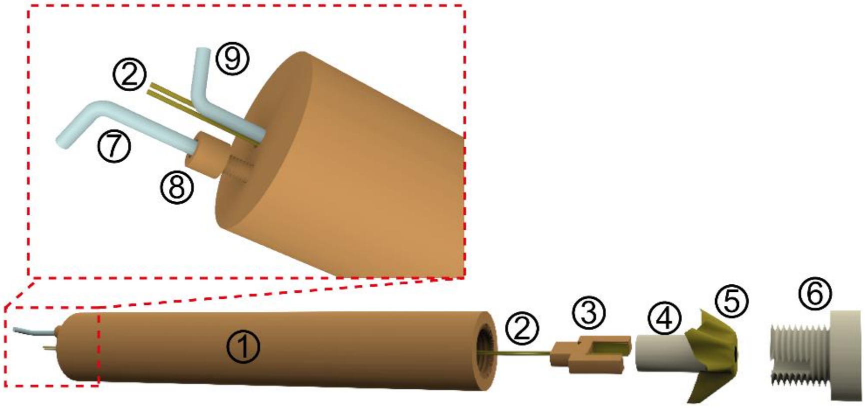

Figure S1. Exploded view of the GAME system, with the top part highlighted in the enlarged image. The GAME is composed of the following parts: 1. PEEK body, 2. Two PTFE-insulated Au wires (connected to the WE and WS, respectively), 3. Au foil-embedded PEEK clip, 4. PTFE cylinder, 5. Au-sputtered PCTE membrane electrode with catalysts, 6. PTFE tip, 7. Gas inlet, 8. PEEK screw to hold the gas inlet tubing, 9. Gas outlet.

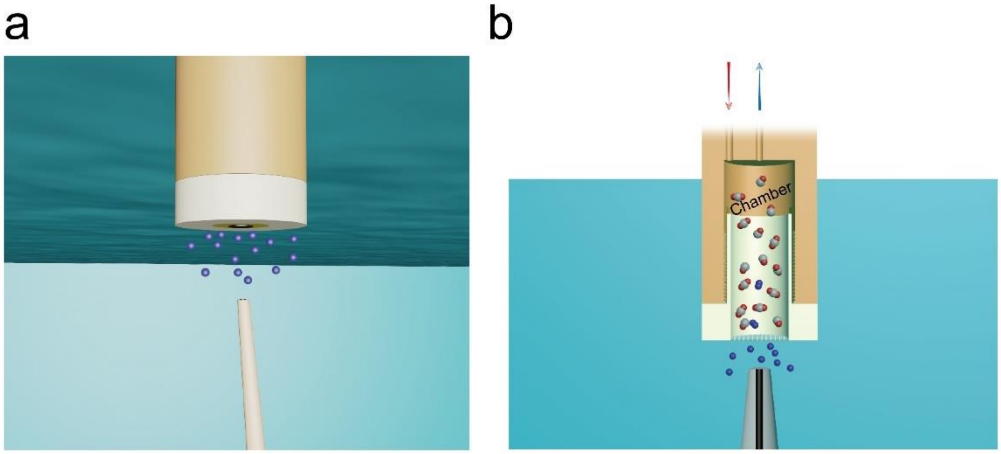

Figure S2. Schematics of the GAME-UME setup. (a) The image shows the very end of the UME positioned in close vicinity to the GAME. (b) The sliced view of the GAME demonstrates the scenarios of mass transport inside the GAME and product detection at the surface of UME. Red and blue arrows indicate the gas inflow and outflow of the GAME, respectively.

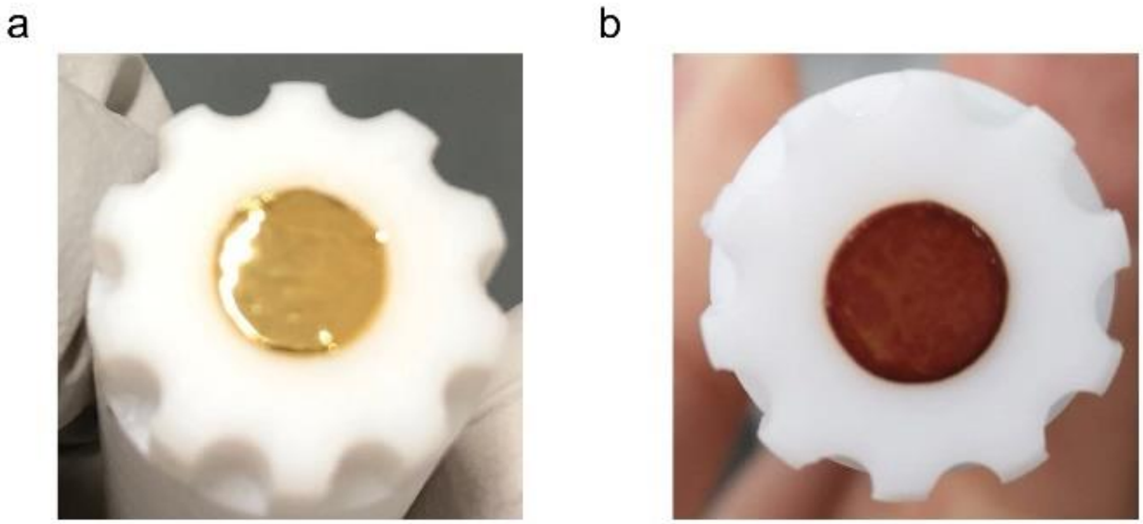

Figure S3. Optical images of the (a) Au/PCTE and (b) Cu-Au/PCTE, respectively.

S2| Onset potential and current efficiency determination

The onset potential of CVs is defined as the potential at which the signal reaches 1% of the highest detectable value.[1-2] The values for the two samples studied under different conditions are summarized in Table S1.

Table S1. Onset potentials values taken from the CVs shown in Figure 3 in the main text.

Sample	Gas atmosphere in the GAME	Onset potential[a] / V vs. RHE
Au/PCTE	N_2	-0.65
	CO_2	-0.62
Cu-Au/PCTE	N_2	-0.69
	CO_2	-0.63

[a] Values are derived from the negative-going scans.

Current efficiency: calculated to evaluate the two competitive reactions (CO_2RR and HER) using the following equation:[3-4]

$$Current\ efficiency = \frac{j_{CO_2} - j_{N_2}}{j_{CO_2}} \times 100\% \qquad (S1)$$

where j_{CO2} and j_{N2} are the current densities obtained in CO_2 and N_2-saturated electrolytes, respectively.

S3| Estimation of ECSA of Au/PCTE

The electrochemical surface area (ECSA) of Au/PCTE was characterised by integrating the reduction peak of gold oxide and also by using the copper underpotential deposition (UPD) approach. For the reduction peak-based method (Figure S4a), a specific charge of 390 μC cm^{-2} was taken for one monolayer of gold[5] and for the copper UPD measurements, a charge of 92.4 μC cm^{-2} was assumed.[6] Note that the potential in Figure S4b is plotted against both Hg/Hg_2SO_4 and Cu/Cu^{2+} (standard potentials: 0.613 V and 0.34 V, respectively[7]). For a typical Au/PCTE sample, the ECSA value of 1.66 cm^2 obtained from the former method corresponds well with 1.53 cm^2 from the latter. However, in the main text, we only report the geometric current densities.

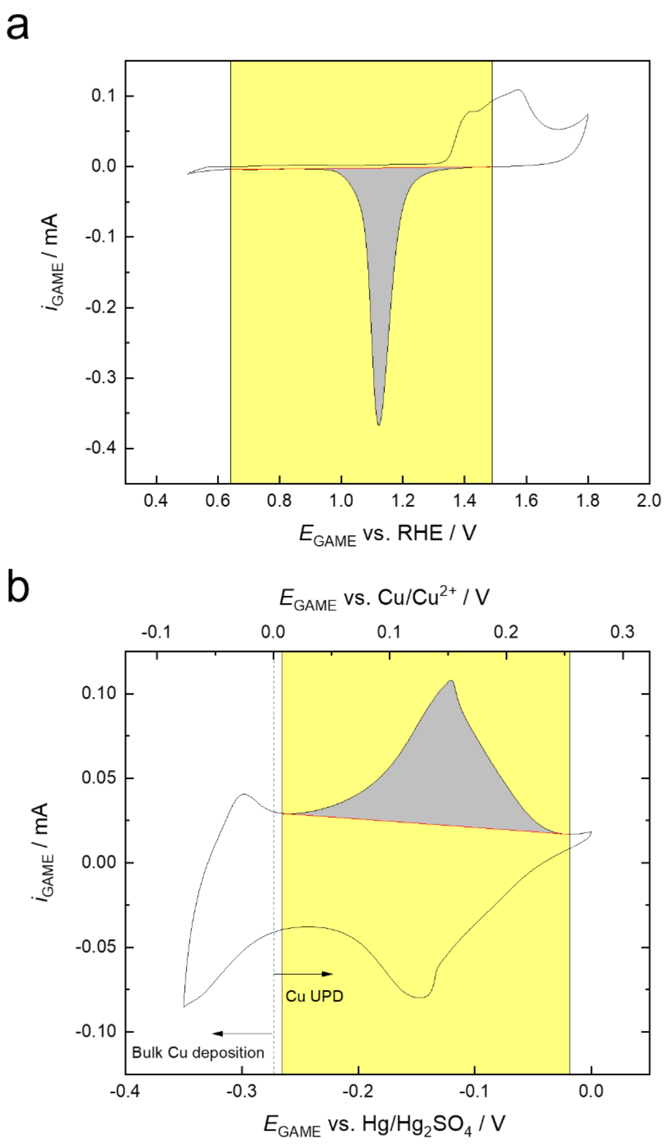

Figure S4. CV of Au/PCTE (a) in 0.5 M H_2SO_4 and (b) in a solution of 0.5 H_2SO_4 containing 0.1 M $CuSO_4$, recorded at 50 mV s^{-1}.

S4| The response time of mass spectrometric signals

In the coupled electrochemical-mass spectrometry setups, it takes some time (i.e., the response/delay time) for the electrochemically generated species to be detected after production. The time can possibly arise from several parts: the flow through the electrolyte, the membrane and the interconnections (e.g., tubings).[8-9] And this needs to be taken into consideration during data analysis in order to correlate the mass spectrometric results with Faradaic currents.

The response time has been usually determined by performing the hydrogen evolution reaction either under steady state or transient conditions.[10-12] For steady state tests, current or potential steps are applied and the electrochemical current and mass ion current are recorded as a function of time. The response time is taken as the time difference when each signal starts to change. However, with respect to transient responses (under cyclic scanning conditions), the response time is reported to be highly dependent on the scan rate and a high scan rate leads to longer response time.[12]

For our system, the steady state measurements, including both constant current and constant potential steps, were first carried out, from which the time constant can be determined by fitting the results using the following equation:[13-14]

$$p = p_{end} + (p_{start} - p_{end})e^{-(t-t_0-t_d)/\tau} \text{ (S2)}$$

where p is the partial pressure for product species, p_{start} and p_{end} are the partial pressures at steady state before and after the current/potential step, respectively, t_0 is the time for step change in Faradaic current or potential, t_{d} is the delay time between the moment of step change in electrochemical signals and the beginning of observable MS responses, and τ is the time constant. As seen from Figure S5 and Table S2, the time constant is generally <8 s and averaged to be 6.4 s (n=8). Similar values are obtained for the increasing and decreasing steps, indicative of the rapid mass transport of product species.

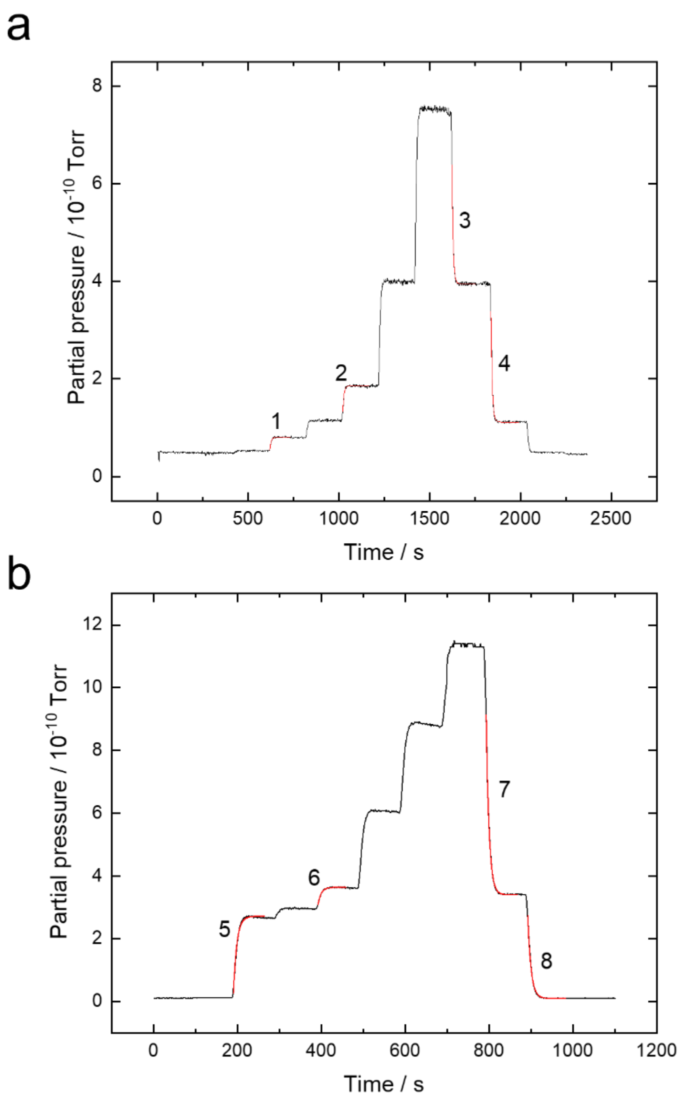

Figure S5. Mass spectrometric signals for H_2 (m/z=2) recorded under (a) current steps and (b) potential steps using a Pt/C-deposited Au/PCTE electrode in 1 M $HClO_4$. The fittings following Eq.(S2) are shown in red.

Table S2. Summary of the parameter values derived from the fittings shown in Figure S5.

Fitting number	τ / s	R^2
1	7.2	0.944
2	6.3	0.958
3	6.4	0.995
4	6.9	0.996
5	8.1	0.992
6	7.5	0.987
7	7	0.999
8	7.9	0.998

Next, linear scan voltammetry (LSV) of Au/PCTE was performed at a rate of 10 mV s^{-1} in 0.5 M $KHCO_3$ to assess the response time under transient conditions, while the mass spectrometric signal was simultaneously recorded. As demonstrated in Figure S6, the response time for H_2 evolution is ~8 s, in good accordance with that seen for static measurements. The chronoamperometry and chronopotentiometry results presented in main text were thus corrected by this value.

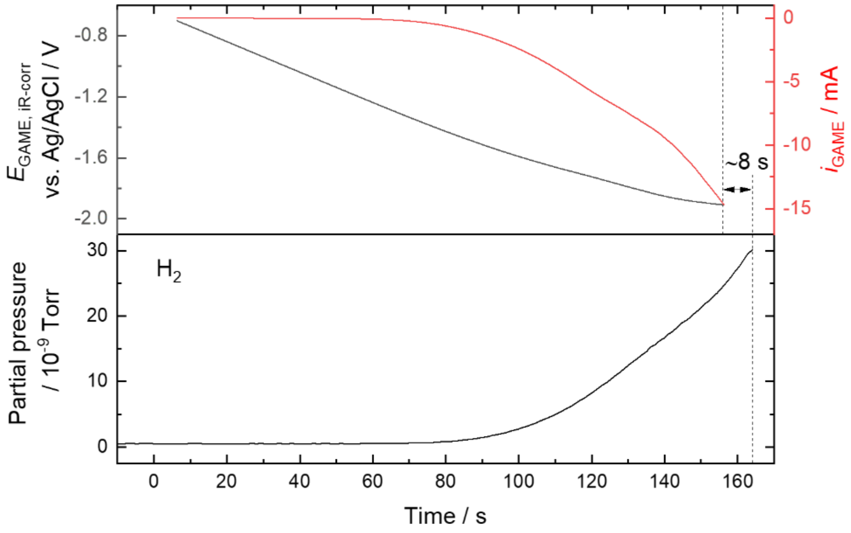

Figure S6. Top panel: Faradaic current and potential profiles during LSV of Au/PCTE recorded in 0.5 M $KHCO_3$ when the GAME is purged with N_2. Scan rate: 10 mV s^{-1}. Bottom panel: corresponding mass spectrometric response for H_2 partial pressure.

S5| Chronoamperometry and MS responses of Au/PCTE

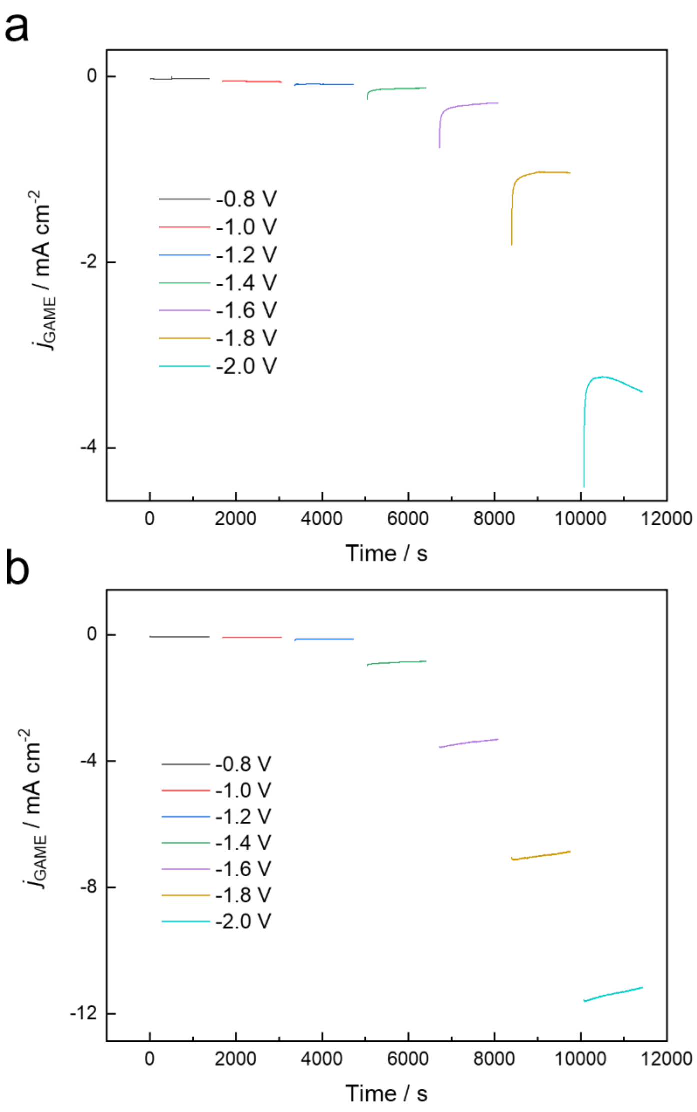

Figure S7. Current responses of the Au/PCTE under N_2 (a) and CO_2 (b) atmosphere, respectively, recorded in 0.1 M $KHCO_3$. Note that the potentials shown in the legend are the values versus Ag/AgCl experimentally applied to the GAME, i.e., without iR corrections.

Figure S8 shows the mass ion currents for H_2 (*m/z*=2), C_2H_4 (*m/z*=26) and CH_4 (*m/z*=15) while N_2 or CO_2 is supplied to the Au/PCTE in the GAME. It can be seen that, in both cases, only H_2 can be detected by mass spectroscopy. Note that CO is likely to be produced as well at the Au/PCTE surface, but this is currently not monitored due to the large overlap with CO_2 fragments.

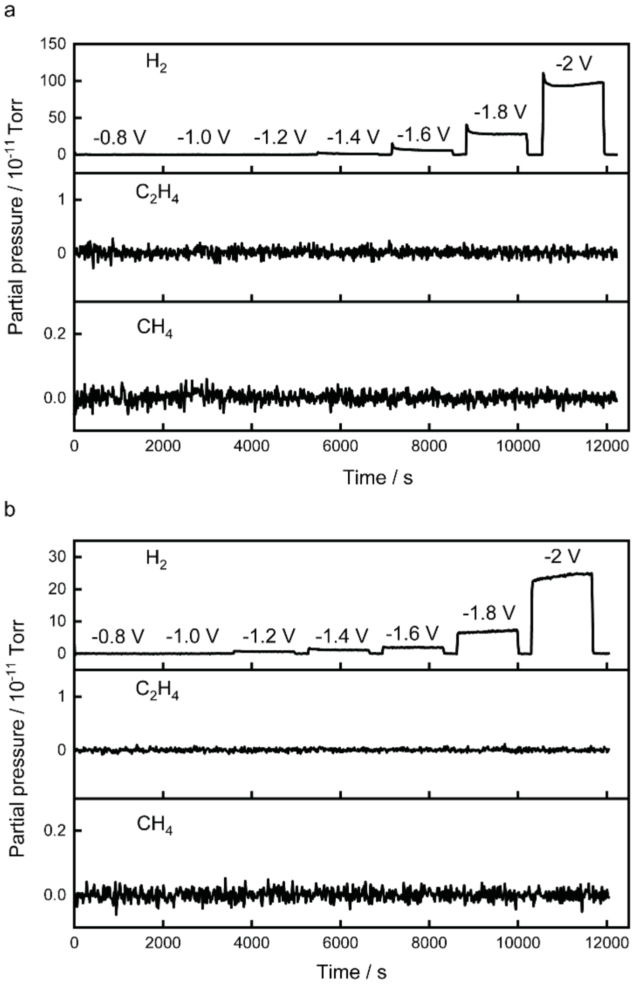

Figure S8. Mass signals for H_2 (*m/z*=2), C_2H_4 (*m/z*=26) and CH_4 (*m/z*=15) simultaneously recorded during chronoamperometry of Au/PCTE in 0.1 M $KHCO_3$, with the GAME supplied with (a) N_2 and (b) CO_2.

S6| Chronoamperometry and chronopotentiometry measurements for the CO_2RR on Cu-Au/PCTE

During the electrochemical measurements, the MS data for the products was recorded. For Cu-Au/PCTE, H_2, C_2H_4, and CH_4 are detected under CO_2. These hydrocarbons are absent during the reaction in N_2, because no exchange equilibrium can be established between gaseous CO_2 and the bicarbonate electrolyte, which provides the source of CO_2 for the reaction.[15-16] The MS data collected from the Cu-Au/PCTE sample is then converted to the molar flow rate (mol s^{-1}) using the equations below:

$$N_i = x_i N = x_i \frac{Pv}{RT} \qquad \text{(S3)}$$

$$x_i = \frac{p_i}{p_{tot}} \qquad \text{(S4)}$$

where N_i is the flux of species i, mol s^{-1}; x_i is the molar fraction of species i (obtained from the mass spectrometry results in %, as the result of partial pressure of i , p_i, divided by the total partial pressure p_{tot}, Eq.(S4)), N is the total flux, mol s^{-1}; and P is the atmospheric pressure, 101325 Pa; v is the volumetric flow rate, 3.67×10^{-7} m^3 s^{-1} (converted from the gas flow of 22 mL min^{-1}); R is the gas constant, 8.314 J K^{-1} mol^{-1} and T is room temperature, 298.15 K. The gas products are assumed to be well mixed. The calculation is exemplified by using the MS data recorded during the chronoamperometry of Cu-Au/PCTE for CO_2RR. In Figure S9, the corrected partial pressures are shown after the calibration with a gas mixture. At 2000 s, the partial pressures for H_2, C_2H_4, CH_4 are 1.38 $\times10^{-9}$, 1.80×10^{-10} and 7.70×10^{-12} Torr, respectively, while that for CO_2 is 2.3×10^{-6} Torr. Therefore, their molar fractions are 0.06%, 0.0078% and 0.00033%, respectively. Since the flow rate of CO_2 into the electrochemical cell is fixed at 22 mL min^{-1}, the total flux N would be 1.5×10^{-5} mol s^{-1}. Then, the molar flow rates for H_2, C_2H_4, CH_4 would be calculated to be ~9, 1.2 and 0.05 nmol s^{-1}, respectively. The full results in molar flow rates are presented in Figure S10a.

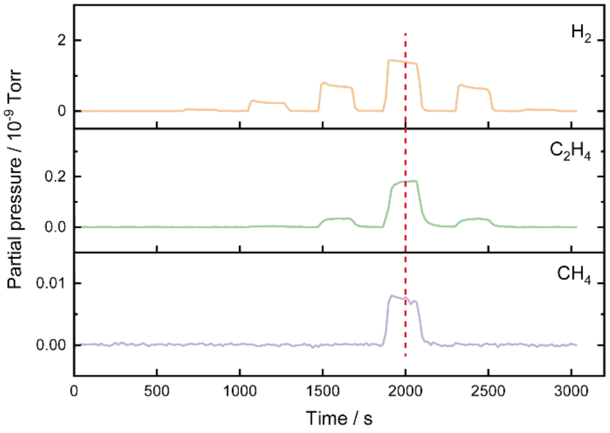

Figure S9. MS responses (in partial pressures corrected by calibration) during chronoamperometry of Cu-Au/PCTE for CO_2RR. The dashed line indicates the data point at 2000 s taken for the conversion calculation.

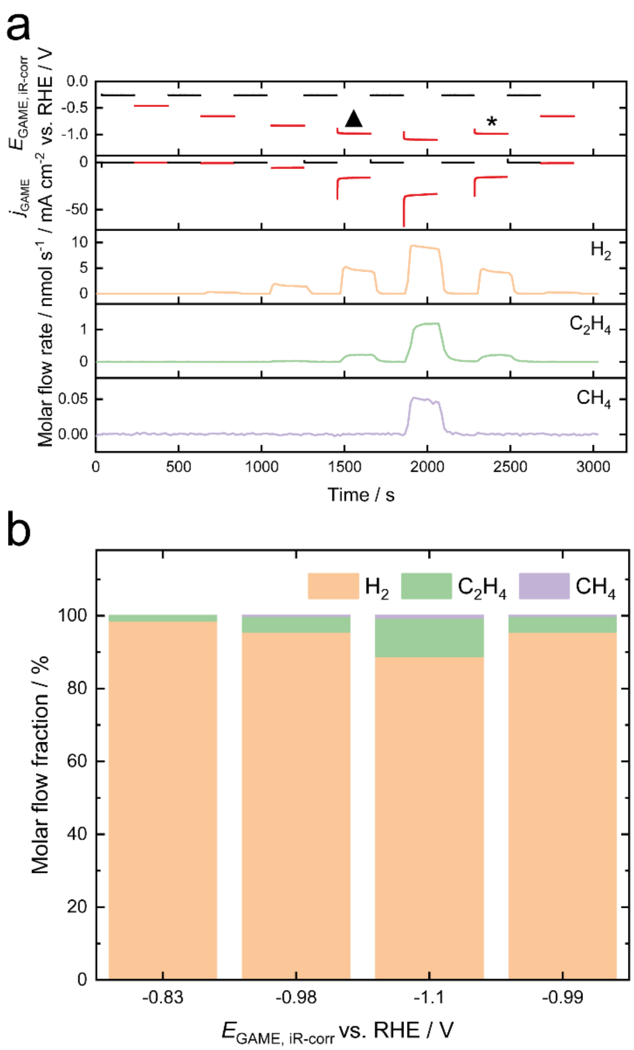

Figure S10. (a) Potential, Faradaic current profiles and corresponding molar flow rates for H_2 (*m/z*=2), C_2H_4 (*m/z*=26) and CH_4 (*m/z*=15) during chronoamperometry of Cu-Au/PCTE for CO_2RR in a solution of 0.5 M $KHCO_3$, under pure CO_2 flow. The potential steps (red) are separated by a step of -0.26 V for 200 s (black) to allow the MS signal to return to baseline. Symbols indicate the potential steps of -0.98 V (▲) and -0.99 V (∗), respectively. (b) Corresponding molar flow fraction values of H_2, C_2H_4 and CH_4 at selective potentials.

As not all CO_2RR products are currently monitored in this setup,[17] the delivery of absolute quantitative information is not realistic and instead the relative product distribution is evaluated by the mass flow fraction (MFF) (Figure S10b). The MFF can be calculated from Eqs. (S5-S7) below:[1, 18]

$$MFF_{H2} = \frac{N_{H2}}{N_{H2}+N_{C2H4}+N_{CH4}} \times 100\ (\%) \qquad \text{(S5)}$$

$$MFF_{C2H4} = \frac{N_{C2H4}}{N_{H2}+N_{C2H4}+N_{CH4}} \times 100\ (\%) \qquad \text{(S6)}$$

$$MFF_{CH4} = \frac{N_{CH4}}{N_{H2}+N_{C2H4}+N_{CH4}} \times 100\ (\%) \qquad \text{(S7)}$$

where N_{H2}, N_{C2H4} and N_{CH4} are mass flow rates for H_2, C_2H_4 and CH_4, respectively.

Note that the MFFs for the three gas species at -0.98 V during the potential-decreasing phase (▲) are very similar to their counterparts at -0.99 V (∗) during the potential-increasing phase (95.6%, 4.3% and 0.01% for H_2, C_2H_4 and CH_4 respectively). This behavior shows the collection efficiency of the GAME is constant during the measurements and there is almost no aging effect over the period.

In the chronopotentiometry measurements, a series of constant current steps (determined from the CV shown in Figure S11) are applied. With the use of this protocol, the possible ohmic drop effects can be easily applied to the recorded potentials and a direct correlation of electrochemical currents and MS responses can thus be provided. Again, the MS signals promptly follows the continuous step changes in Faradaic currents (Figure S12), the same currents in the ascending steps and the descending steps show almost identical mass flow rates (also see Figure S13). It is also worth mentioning that all the gases studied herein are evolved at the same time, with no obvious delay effect arising from mass transport variation seen in our system.[19-20]

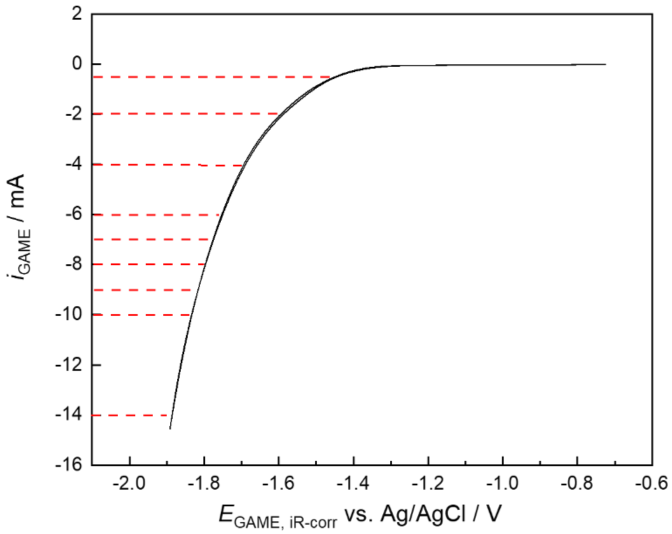

Figure S11. CV of Cu-Au/PCTE for CO_2RR recorded at 5 mV s^{-1} in 0.5 M $KHCO_3$.

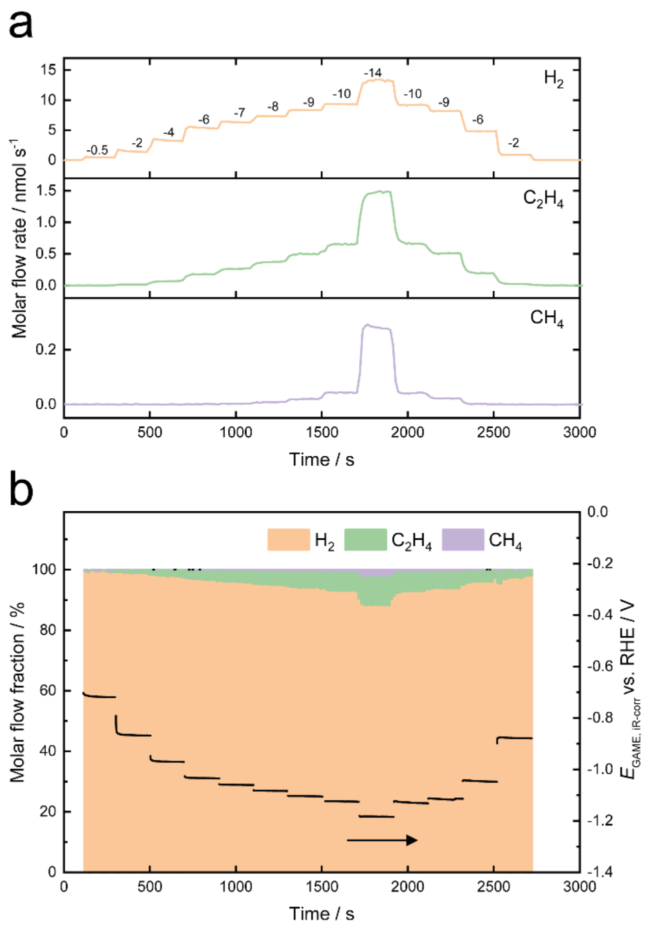

Figure S12. (a) Molar flow rates for H_2 (*m/z*=2), C_2H_4 (*m/z*=26) and CH_4 (*m/z*=15), recorded during chronopotentiometry of Cu-Au/PCTE for CO_2RR in a solution of 0.5 M $KHCO_3$. The applied currents are indicated by the numbers (unit: mA). (b) Corresponding molar flow fraction values of H_2, C_2H_4 and CH_4 and potential responses under a sequence of current steps.

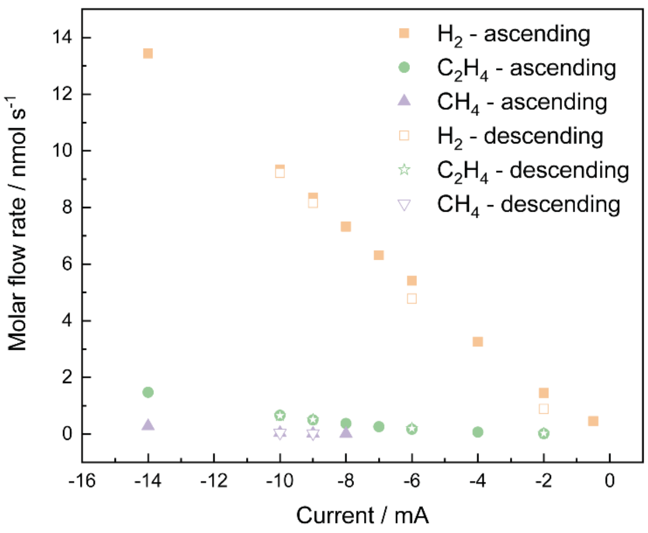

Figure S13. Plot of MS response as a function of electrochemical current during chronopotentiometry.

S7| Electrochemical characterisation of the UME probe

The Pt UME (d= 25 µm) was first electrochemically characterized in 0.5 M H_2SO_4, demonstrating typical features of Pt electrodes (Figure S14a). The Pt UME was also studied in 1 mM $K_4Fe(CN)_6$ and 0.1 M KCl using cyclic voltammetry, as shown in Figure S14b. The limiting current, i_{lim}, can be estimated from Eq. (S8):[7]

$$i_{lim} = 4nFDcr \qquad \text{(S8)}$$

Where n is the number of electrons transferred, D is the diffusion coefficient of ferrocyanide (6.66×10^{-6} $cm^2\ s^{-1}$),[21] c is the concentration, and r is the effective radius of the Pt electrode (12.5 µm). From the CV shown in Figure S14b, a limiting current of ~3.1 nA is observed, in good agreement with the theoretically expected current.

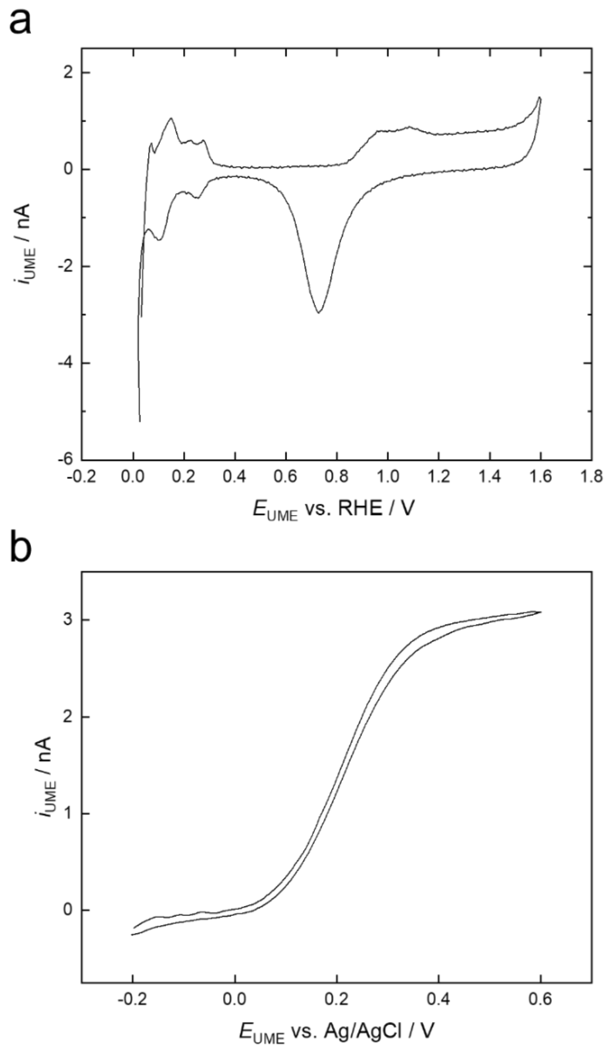

Figure S14. CVs of Pt UME in (a) 0.5 M H_2SO_4 scanned at 50 mV s^{-1} and (b) 1 mM $K_4Fe(CN)_6$ in 0.1 M KCl, recorded at a scan rate of 1 mV s^{-1}.

Figures15 shows an optical micrograph of the UME tip close to the GAME electrode.

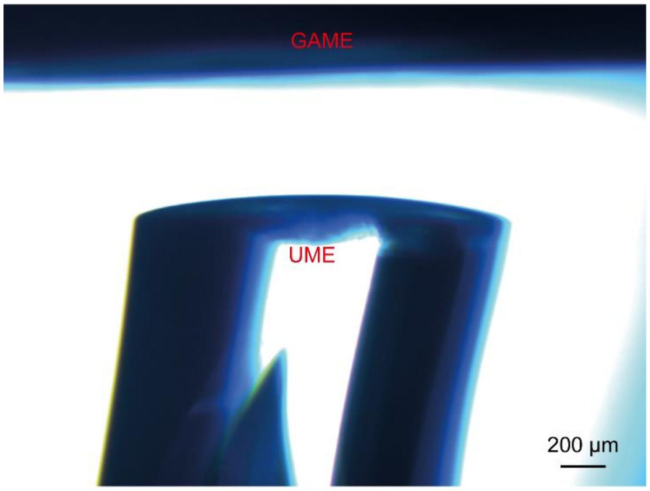

Figure S15. Optical image of the Pt UME placed adjacent to the surface of the GAME in a solution of 0.1 M $KHCO_3$.

S8| Electrical cross-talk effect in the current configuration

It is noteworthy that in a four-electrode system (e.g., SECM and RRDE), there is an electrical cross-talk effect between the two working electrodes (i.e., the GAME and the UME) caused by the shared current route, and this can introduce a shift in the potentials at both electrodes.[22-26] To solve this issue, we performed the iR corrections based on the following circuit (Figure S16).

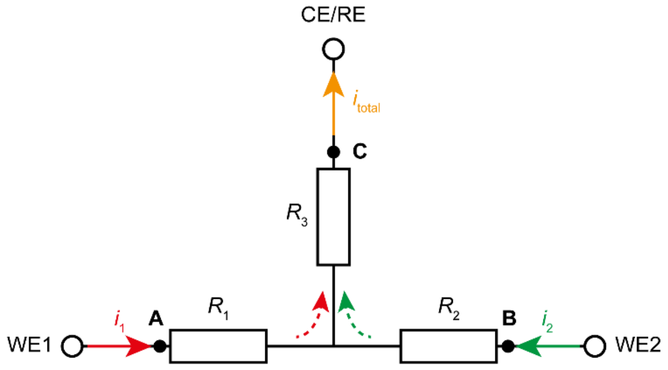

Figure S16. Electrical equivalent circuit for a four-electrode system. Points A, B and C represent the position for the working electrode 1 (WE1), the working electrode 2 (WE2) and the reference electrode, respectively.

The total current flowing through resistance R_3 would be:

$$i_{total} = i_1 + i_2 \qquad \text{(S9)}$$

where i_1 and i_2 are the currents at the WE1 and WE2, respectively.

The potentials applied to the two working electrode both suffer ohmic drops (the voltage between point A and C for WE1, and the voltage between point B and C for WE2):

$$E_{WE1,iR-corrected} = E_{WE1,applied} - (i_1R_1 + i_{total}R_3) = E_{WE1,applied} - (i_1R_1 + i_1R_3 + i_2R_3) \text{ (S10)}$$

$$E_{WE2,iR-corrected} = E_{WE2,applied} - (i_2R_2 + i_{total}R_3) = E_{WE2,applied} - (i_2R_2 + i_1R_3 + i_2R_3) \text{ (S11)}$$

In our work, we took the uncompensated resistance R_s as the R_3 to simplify the case studied and correspondingly R_1=R_2 ≈0. Then, Eqs. (S10-S11) can be transformed to:

$$E_{WE1,iR-corrected} = E_{WE1,applied} - (i_1 + i_2)R_s \qquad \text{(S12)}$$

$$E_{WE2,iR-corrected} = E_{WE2,applied} - (i_1 + i_2)R_s \qquad \text{(S13)}$$

S9| Pt UME responses during chronoamperometry

The CVs of the Pt UME were then iR-corrected using the protocol presented above, as illustrated in Figure S17. Figure S18 displays the replicates of UME CVs when a range of Au/PCTE electrodes were used on different days and held at different potentials under CO_2. Note that the UME CVs may slightly vary, as the position of the UME in relation to the GAME is difficult to be kept the same for all the measurements. In general, a good reproducibility of the data is seen, highlighting the strength of the UME for the detection of CO_2RR products species in the electrolyte. Figure S19 compares the GAME current plotted against the corrected potential for both CV and chronoamperometric experiments.

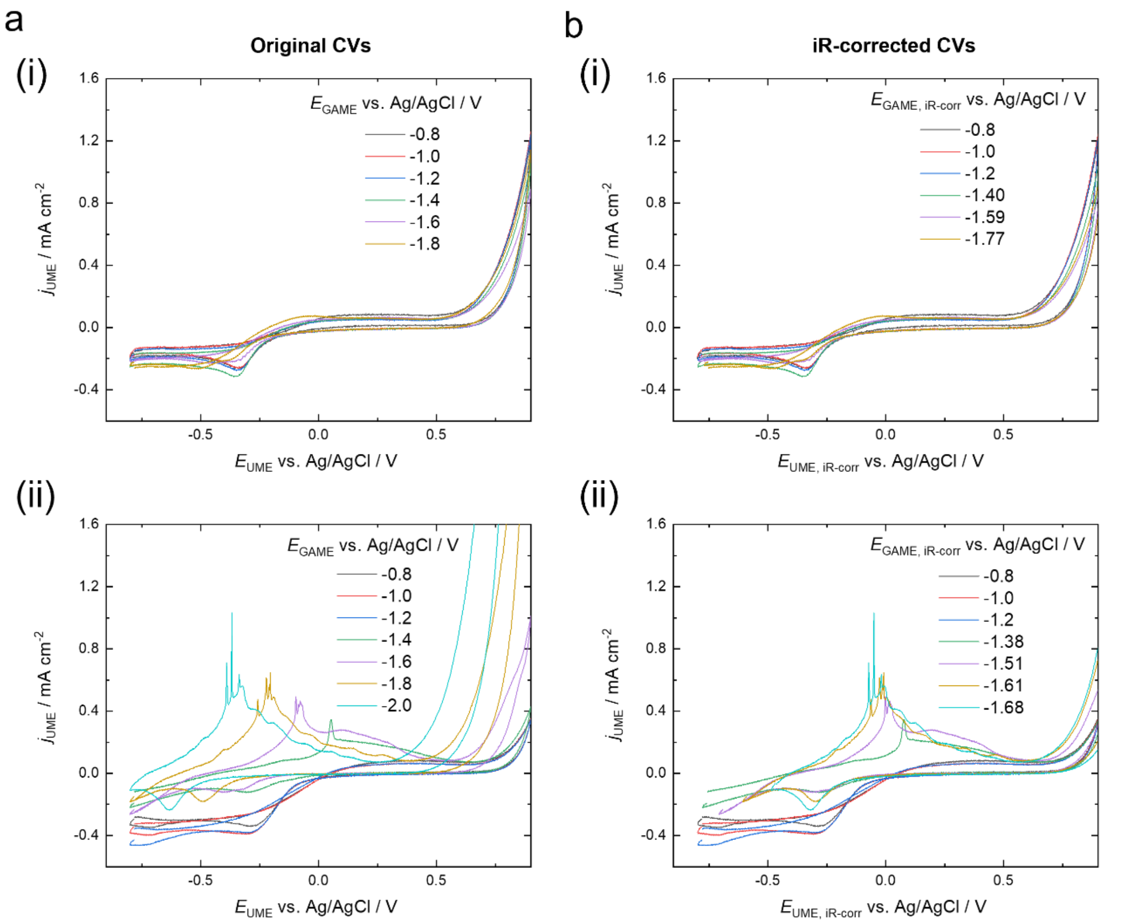

Figure S17. (a) As-obtained and (b) iR-corrected CVs of the Pt UME in 0.1 M $KHCO_3$ saturated with N_2 while a series of constant potential steps (indicated by the values in the legends; on Ag/AgCl scale) are applied to the Au/PCTE of the GAME supplied with a gas flow of (i) N_2 and (ii) CO_2, respectively.

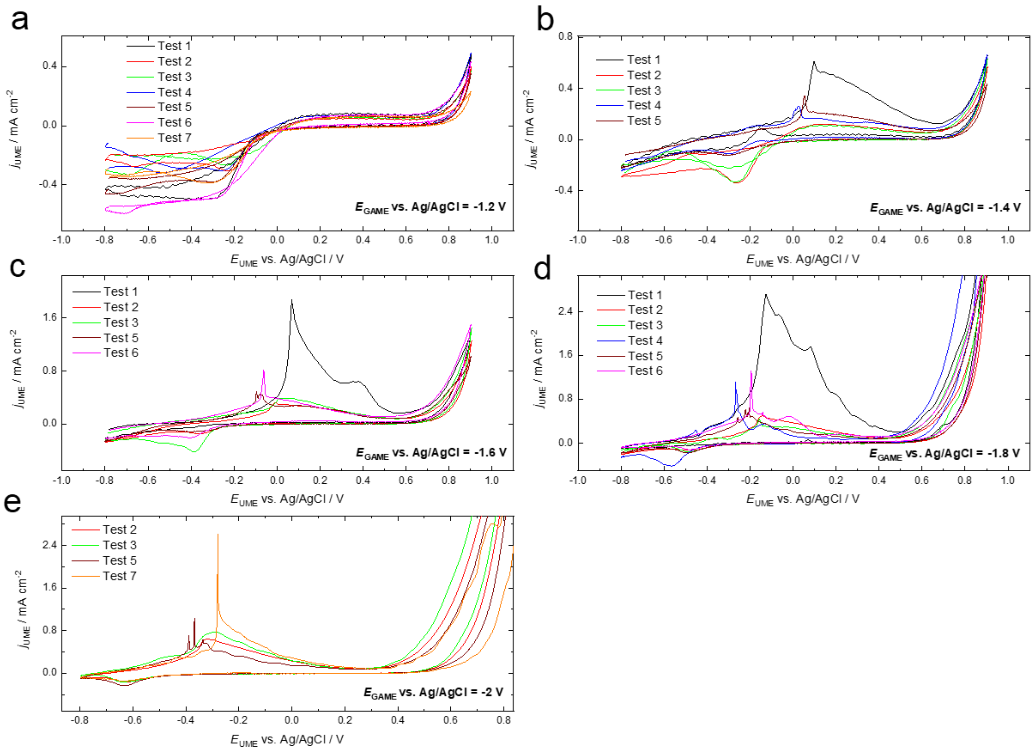

Figure S18. Replicates of UME CVs from a range of Au/PCTE samples recorded at a potential of (a)-1.2, (b) -1.4, (c)-1.6, (d)-1.8 and (e)-2.0 V applied to the GAME purged with CO_2, respectively. Note that all the potentials are shown without iR correction.

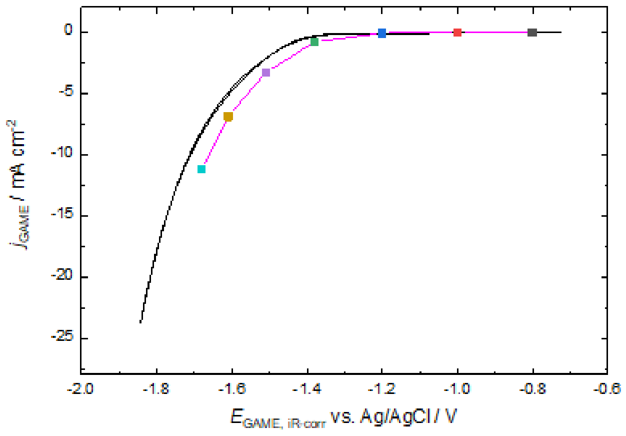

Figure S19. Comparison of current densities recorded by CV (black line; also shown in main text) and chronoamperometry measurements (magenta; also shown in Figure 6b of main text) obtained on two different Au/PCTE samples under CO_2 atmosphere, respectively.

S10| Estimation of pH during the electrolysis under constant potential steps on the GAME

The pH in the vicinity of the electrode is reported to change significantly during the CO_2RR in some systems, particularily with large-area electrodes and under high current densities. Therefore, this aspect needs to be taken into consideration, not only for the correction of corresponding potential shift, but also for the optimisation of the reaction system design. The pH change has been widely estimated by modelling and calculations,[27-28] but there are also some papers where this was experimentally measured.[29-30]

In our work, we use the change of the reduction peak of Pt oxide on the UME as as an indicator of the pH shift (*cf*. Figure S17), as shown in Eqs.(S14-S15). The pH of 9.2 for the N_2-saturated solution was taken as the start value for both the electrolysis in N_2 and CO_2. This assumption was made based on the following reasons: the bulk electrolyte was extensively sparged with N_2 prior to the measuments and maintained in the air-tight electrochemical cell; the GAME electrode (geometric area: 0.35 cm^2) was much smaller compared to the domain of the bulk electrolyte, which could only induce a local pH change; the gas change (from N_2 to CO_2) only occurred in the GAME; a rest time of ~310 s was applied in between the potential steps on the GAME, and CO_2RR was started an hour after the measurements under N_2, allowing for the replienshmentof solution to the local environment surrounding the GAME.

$$PtO + H_2O + 2e^- \rightleftharpoons Pt + 2OH^- \quad \text{(S14)}$$
$$\Delta pH = \frac{\Delta E}{0.059} \quad \text{(S15)}$$

where ΔpH is the pH change from the electrolysis at one potential to another, and ΔE is the corresponding variation in peak potentials.

The calculated results are demonstrated in Table S3. Under N_2, with the decrease of the potential applied to the GAME, the local pH slightly increases and changes from 9.2 to 9.6 after the electrolysis of potential steps. In contrast, the pH change is more significant during the electrolysis under the atmosphere of CO_2 over the high overpotential region and a shift of 0.8 is seen after the measurements.

Table S3. Estimated pH changes under a series of constant potentials applied to the GAME (with iR corrections) and corresponding current densities, under N_2 and CO_2 conditions.

GAME potential in N_2 vs Ag/AgCl (iR corrected) / V	GAME current density in N_2 / mA cm^{-2}	Estimated pH	GAME potential in CO_2 vs Ag/AgCl (iR corrected) / V	GAME current density in CO_2 / mA cm^{-2}	Estimated pH
-0.8	-0.025	9.2	-0.8	-0.051	9.2
-1	-0.055	9.23	-1	-0.074	9.25
-1.2	-0.085	9.27	-1.2	-0.12	9.3
-1.4	-0.13	9.39	-1.38	-0.85	9.42
-1.59	-0.29	9.42	-1.51	-3.34	9.57
-1.77	-1.03	9.45	-1.61	-6.94	9.61
-1.91	-3.34	9.56	-1.68	-11.26	10.01

S11| UME CV during chronoamperometric measurements on the GAME

Note that in some cases, the UME CV can slightly differ in the cathodic scan by showing a weak oxidation peak (Figure S20).

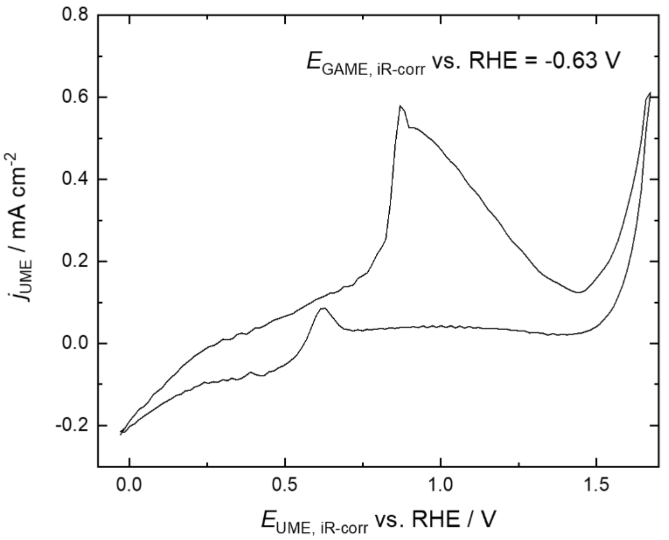

Figure S20. UME CV recorded at 5 mV s^{-1} in N_2-saturated 0.1 M $KHCO_3$, while -1.4 V vs Ag/AgCl (-0.63 V vs RHE) was applied to the Au/PCTE GAME.

S12| NMR spectra of solutions after electrolysis

The electrolyte after 1 h of electrolysis at different potentials was characterised by NMR. From Figure S21, it can be seen that there is one well-defined peak at ~8.35 in the spectra, indicating formate is produced in the solution during the CO_2RR on Au/PCTE, in line with literature.[1, 31]

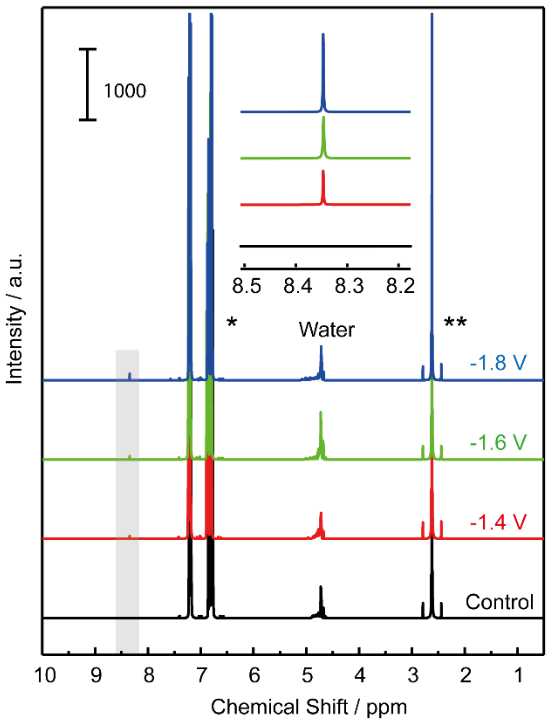

Figure S21. ^1H NMR spectra of 0.1 M $KHCO_3$ before and after 1 h of CO_2RR electrolysis at different potentials (vs Ag/AgCl) on Au/PCTE. Note that phenol (*) and DMSO (**) were used as the internal standards.

References

1. Todoroki, N.; Tei, H.; Tsurumaki, H.; Miyakawa, T.; Inoue, T.; Wadayama, T. Surface Atomic Arrangement Dependence of Electrochemical CO_2 Reduction on Gold: Online Electrochemical Mass Spectrometric Study on Low-Index Au(hkl) Surfaces. *ACS Catal* **2019,** *9* (2), 1383-1388.
2. Wang, X. L.; de Araujo, J. F.; Ju, W.; Bagger, A.; Schmies, H.; Kuhl, S.; Rossmeisl, J.; Strasser, P. Mechanistic Reaction Pathways of Enhanced Ethylene Yields during Electroreduction of CO_2-CO Co-Feeds on Cu and Cu-Tandem Electrocatalysts. *Nat. Nanotechnol.* **2019,** *14* (11), 1063-1070.
3. Chen, S.; Chen, A. C. Electrochemical Reduction of Carbon Dioxide on Au Nanoparticles: An in Situ FTIR Study. *J. Phys. Chem. C* **2019,** *123* (39), 23898-23906.
4. Hussain, J.; Jonsson, H.; Skulason, E. Calculations of Product Selectivity in Electrochemical CO_2 Reduction. *ACS Catal.* **2018,** *8* (6), 5240-5249.
5. Goyal, A.; Marcandalli, G.; Mints, V. A.; Koper, M. T. M. Competition between CO_2 Reduction and Hydrogen Evolution on a Gold Electrode under Well-Defined Mass Transport Conditions. *J. Am. Chem. Soc.* **2020,** *142* (9), 4154-4161.
6. Mariano, R. G.; McKelvey, K.; White, H. S.; Kanan, M. W. Selective Increase in CO_2 Electroreduction Activity at Grain-Boundary Surface Terminations. *Science* **2017,** *358* (6367), 1187-1191.
7. Allen J. Bard; Faulkner, L. R., *Electrochemical Methods: Fundamental and Applications*. 2nd ed.; John Wiley & Sons: New York, 2001.
8. Skou, E.; Munk, J. A Simplified Set-up for Electrochemical Mass-Spectrometry and the Use of a Gold Substrate for the Investigation of Porous-Electrode Materials. *J. Electroanal. Chem.* **1994,** *367* (1-2), 93-98.
9. Wonders, A. H.; Housmans, T. H. M.; Rosca, V.; Koper, M. T. M. On-line Mass Spectrometry System for Measurements at Single-Crystal Electrodes in Hanging Meniscus Configuration. *J. Appl. Electrochem.* **2006,** *36* (11), 1215-1221.
10. Wasmus, S.; Samms, S. R.; Savinell, R. F. Multipurpose Electrochemical Mass-Spectrometry - a New Powerful Extension of Differential Electrochemical Mass-Spectrometry. *J. Electrochem. Soc.* **1995,** *142* (4), 1183-1189.
11. Castro-Castillo, C.; Armijo, F.; Isaacs, M.; Pastor, E.; Garcia, G. Flow Injection Analysis Coupled with Differential Electrochemical Mass Spectrometry for Hydrogen Detection and Quantification. *Electrochem. Commun.* **2020,** *118*, 106809.
12. Perez-Rodriguez, S.; Corengia, M.; Garcia, G.; Zinola, C. F.; Lazaro, M. J.; Pastor, E. Gas Diffusion Electrodes for Methanol Electrooxidation Studied by a New DEMS Configuration: Influence of the Diffusion Layer. *Int. J. Hydrogen Energy* **2012,** *37* (8), 7141-7151.
13. Zhang, G. H.; Kucernak, A. Gas Accessible Membrane Electrode (GAME): A Versatile Platform for Elucidating Electrocatalytic Processes Using Real-Time and in Situ Hyphenated Electrochemical Techniques. *ACS Catal.* **2020,** *10* (17), 9684-9693.
14. Reischl, P.; Beaucage, C. B. Time Constant and Transport Delay Determination of the Gas Analyzer. *Math. Model.* **1986,** *7* (9-12), 1613-1620.
15. Dunwell, M.; Lu, Q.; Heyes, J. M.; Rosen, J.; Chen, J. G. G.; Yan, Y. S.; Jiao, F.; Xu, B. J. The Central Role of Bicarbonate in the Electrochemical Reduction of Carbon Dioxide on Gold. *J. Am. Chem. Soc.* **2017,** *139* (10), 3774-3783.
16. Zhu, S. Q.; Jiang, B.; Cai, W. B.; Shao, M. H. Direct Observation on Reaction Intermediates and the Role of Bicarbonate Anions in CO_2 Electrochemical Reduction Reaction on Cu Surfaces. *J. Am. Chem. Soc.* **2017,** *139* (44), 15664-15667.
17. Roberts, F. S.; Kuhl, K. P.; Nilsson, A. High Selectivity for Ethylene from Carbon Dioxide Reduction over Copper Nanocube Electrocatalysts. *Angew. Chem. Int. Ed.* **2015,** *54* (17), 5179-5182.
18. Reske, R.; Duca, M.; Oezaslan, M.; Schouten, K. J. P.; Koper, M. T. M.; Strassert, P. Controlling Catalytic Selectivities during CO_2 Electroreduction on Thin Cu Metal Overlayers. *J. Phys. Chem. Lett.* **2013,** *4* (15), 2410-2413.

19. Grote, J. P.; Zeradjanin, A. R.; Cherevko, S.; Mayrhofer, K. J. J. Coupling of a Scanning Flow Cell with Online Electrochemical Mass Spectrometry for Screening of Reaction Selectivity. *Rev. Sci. Instrum.* **2014,** *85*, 104101.
20. Khanipour, P.; Loffler, M.; Reichert, A. M.; Haase, F. T.; Mayrhofer, K. J. J.; Katsounaros, I. Electrochemical Real-Time Mass Spectrometry (EC-RTMS): Monitoring Electrochemical Reaction Products in Real Time. *Angew. Chem. Int. Ed.* **2019,** *58* (22), 7273-7277.
21. Konopka, S. J.; McDuffie, B. Diffusion Coefficients of Ferri- and Ferrocyanide Ions in Aqueous Media, Using Twin-Electrode Thin-Layer Electrochemistry. *Anal. Chem.* **1970,** *42* (14), 1741-1746.
22. Vesztergom, S.; Barankai, N.; Kovács, N.; Ujvári, M.; Broekmann, P.; Siegenthaler, H.; Láng, G. G. Electrical Cross-Talk in Rotating Ring–Disk Experiments. *Electrochem. Commun.* **2016,** *68*, 54-58.
23. Trinh, D.; Maisonhaute, E.; Vivier, V. Electrical Cross-Talk in Transient Mode of Scanning Electrochemical Microscopy. *Electrochem. Commun.* **2012,** *16* (1), 49-52.
24. Shabrang, M.; Bruckenstein, S. Compensation of Ohmic Potential Interactions Occurring at Ring-Disk Electrodes. *J. Electrochem. Soc.* **1975,** *122* (10), 1305-1311.
25. Shabrang, M.; Bruckenstein, S. Equivalent Circuit for the Uncompensated Resistances Occurring at Ring ‐ Disk Electrodes. *J. Electrochem. Soc.* **1974,** *121* (11), 1439-1444.
26. Vesztergom, S.; Barankai, N.; Kovacs, N.; Ujvari, M.; Siegenthaler, H.; Broekmann, P.; Lang, G. G. Electrical Cross-Talk in Four-Electrode Experiments. *J. Solid State Electrochem.* **2016,** *20* (11), 3165-3177.
27. Gupta, N.; Gattrell, M.; MacDougall, B. Calculation for the Cathode Surface Concentrations in the Electrochemical Reduction of CO_2 in $KHCO_3$ Solutions. *J. Appl. Electrochem.* **2006,** *36* (2), 161-172.
28. Jouny, M.; Luc, W.; Jiao, F. High-rate Electroreduction of Carbon Monoxide to Multi-Carbon Products. *Nat. Catal.* **2018,** *1* (10), 748-755.
29. Li, J.; Chen, G.; Zhu, Y.; Liang, Z.; Pei, A.; Wu, C.-L.; Wang, H.; Lee, H. R.; Liu, K.; Chu, S.; Cui, Y. Efficient Electrocatalytic CO_2 Reduction on a Three-Phase Interface. *Nat. Catal.* **2018,** *1*, 592–600.
30. Zhang, F.; Co, A. C. Direct Evidence of Local pH Change and the Role of Alkali Cation during CO_2 Electroreduction in Aqueous Media. *Angew. Chem. Int. Ed.* **2020,** *59* (4), 1674-1681.
31. Hori, Y.; Wakebe, H.; Tsukamoto, T.; Koga, O. Electrocatalytic Process of CO Selectivity in Electrochemical Reduction of CO_2 at Metal-Electrodes in Aqueous-Media. *Electrochim. Acta* **1994,** *39* (11-12), 1833-1839.
